# Fine mapping of the *Cepaea nemoralis* shell colour and mid-banded loci using a high-density linkage map

**DOI:** 10.1038/s41437-023-00648-z

**Published:** 2023-09-27

**Authors:** Margrethe Johansen, Suzanne Saenko, Menno Schilthuizen, Wellcome Sanger Institute Tree of Life Programme, Matthew Berriman, Richard Durbin, Mara Lawniczak, Sarah Reeks, Kerstin Howe, Nancy Holroyd, Victoria McKenna, Haoyu Niu, Radka Platte, Caroline Howard, Raquel Amaral, Andy Griffiths, Haddijatou Mbye, Graeme Oatley, Liam Prestwood, Filipa Sampaio, Edel Sheerin, Michelle Strickland, Maja Todorovic, Shane A. McCarthy, Ksenia Krasheninnikova, Marcela Uliano-Silva, Jonathan Wood, Will Chow, Joanna Collins, Camilla Eldridge, Michael Paulini, Sarah Pelan, Damon-Lee Pointon, Ying Sims, James Torrance, Alan Tracey, Nikki Chapman, Sinead Calnan, Ken Haug, Robina Heathcote, Chloe Leech, Jack Monaghan, Matthieu Muffato, Sophie Potter, Lauma Ramona, Jonathan Threlfall, Andrew Varley, Amit Vishwakumar, Max Brown, Rich Challis, Pablo Gonzalez, Manuela Kieninger, Erna King, Sujai Kumar, Chris Laumer, Lewis Stevens, Emmelien Vancaester, Claudia Weber, Eerik Aunin, Adam Reid, Mark Blaxter, Angus Davison

**Affiliations:** 1 School of Life Sciences, University Park, University of Nottingham, Nottingham, NG7 2RD UK; 2 Evolutionary Ecology, Naturalis Biodiversity Center, Leiden, 2333CR The Netherlands; 3 Animal Sciences, Institute of Biology Leiden, Leiden University, Leiden, 2333BE The Netherlands; 4 Wellcome Sanger Institute, Wellcome Genome Campus, Hinxton, Cambridgeshire CB10 1SA UK

**Keywords:** Genetic linkage study, Genetic markers, Inbreeding, Evolutionary theory

## Abstract

Molluscs are a highly speciose phylum that exhibits an astonishing array of colours and patterns, yet relatively little progress has been made in identifying the underlying genes that determine phenotypic variation. One prominent example is the land snail *Cepaea nemoralis* for which classical genetic studies have shown that around nine loci, several physically linked and inherited together as a ‘supergene’, control the shell colour and banding polymorphism. As a first step towards identifying the genes involved, we used whole-genome resequencing of individuals from a laboratory cross to construct a high-density linkage map, and then trait mapping to identify 95% confidence intervals for the chromosomal region that contains the supergene, specifically the colour locus (*C*), and the unlinked mid-banded locus (*U*). The linkage map is made up of 215,593 markers, ordered into 22 linkage groups, with one large group making up ~27% of the genome. The *C* locus was mapped to a ~1.3 cM region on linkage group 11, and the *U* locus was mapped to a ~0.7 cM region on linkage group 15. The linkage map will serve as an important resource for further evolutionary and population genomic studies of *C. nemoralis* and related species, as well as the identification of candidate genes within the supergene and for the mid-banding phenotype.

## Introduction

Patterns and colours are traits that can underpin and drive evolutionary and ecological adaptation. In consequence, the pigments and the underlying genetic pathways that produce colour and pattern have been investigated in a wide variety of animals. In vertebrates, the *agouti* locus regulates coat colour pigmentation in mice and other mammals such as the silver fox and sheep (Bultman et al. 1992; Parsons et al. 1999; Vage et al. 1997). In insects, there is a substantial understanding of the genetics of colour, such as the involvement of *cortex* in wing colour patterning in *Heliconius* butterflies and the peppered moth *Biston betularia* (Hof et al. 2016; Nadeau et al. 2016) and the *yellow* gene in *Drosophila* (Biessman 1985; Waddington 1942; Wittkopp et al. 2002).

In comparison, the genetics of molluscan colour and pattern have been relatively neglected. Mollusc shells exhibit an astonishing diversity in colours and pattern, drawing the interest of collectors and scientists for centuries. There has been a long-standing interest in using the inherited colour and pattern variation in snail shells to understand evolution, with most studies using the classic model species the grove snail *Cepaea nemoralis* and the sister taxon *C. hortensis* (Cain et al. 1960; Cain et al. 1990; Cain and Sheppard 1954; Clarke and Murray 1969; Cook 2017; Davison 2002; Jones et al. 1977; Murray and Clarke 1976; Ochman et al. 1983; Silvertown et al. 2011), and processes such as speciation (Butlin et al. 2008; Chiba 2002; Clarke and Murray 1969; Jones et al. 1980). However, there are no well-worked genetic model species, and as an added difficulty, molluscs do not have the standard toolkit of molecular biology available to work on them, with only limited ability to apply RNAi or gene-editing methods (Abe and Kuroda 2019; Albertin et al. 2022; Perry and Henry 2015). Molluscs also generally have large, repetitive genomes, meaning that genome assembly is more difficult, resource intensive and expensive (Adema 2021; Davison and Neiman 2021).

In molluscs that have an aquacultural or economic interest, certain colours of shells are often considered to be ‘high quality’, are preferred by consumers, and thus attract higher market prices. For example, golden-shelled specimens of the Pacific oyster *Crassostrea gigas* reach higher prices (Nell 2001), as do some colour morphs of the hard clam *Mercenaria mercenaria* (Hu et al. 2019) and the Bay scallop *Argopecten irradians* (Qin et al. 2007). The economic importance of these species means that some effort has been put into understanding the genetics of their pigmentation (Ge et al. 2015; Hu et al. 2019; Qin et al. 2007; Zhong et al. 2014) for deployment in marker-assisted breeding programs (Hollenbeck and Johnston 2018; Jiao et al. 2014; Stenger et al. 2021; Zhao et al. 2017). However, even in these relatively well-studied species, there is still a minimal understanding of the molecular underpinning of the genetic regulatory networks responsible for producing and controlling variation in shell colour.

From the available data, it is apparent that the genes and mechanisms responsible for pigmentation in mollusc shells are varied. For example, the gene *Has-sometsuke*, previously shown to be associated with shell pigmentation pattern in the tropical abalone *Haliotis asinine* (Jackson et al. 2006), is not present in the shell-forming proteome of *C. nemoralis* (Mann et al. 2014). Thus, to build a general model of mollusc shell colour and patterning genetics, it will be necessary to define the systems at play in a wide range of species across molluscan classes.

**Fig. 1:**
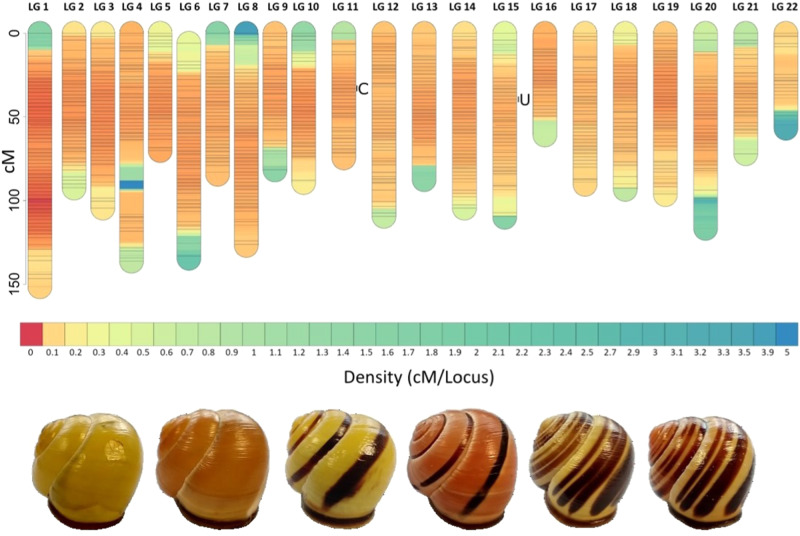
Linkage map showing the density of markers across all chromosomes and approximate position of mapped *C* and *U* loci. Black lines indicate marker positions, and colours show increasing marker density, reflecting the number of markers at each map location, from least dense (blue) to most dense (red). Also shown are exemplar *C. nemoralis* shells, from left: yellow unbanded, pink unbanded, yellow mid-banded, pink mid-banded, yellow five-banded, and pink five-banded.

In *Cepaea*, the shell ground colour is generally categorised as either yellow, pink or brown (Cain and Sheppard 1950; Jones et al. 1977), although recent investigations using spectrophotometry and psycho-physical modelling of avian visual systems have confirmed the long-suspected view that the colour variation is continuously distributed (Davison et al. 2019). As well as variation in the ground colour, shells are also patterned with zero to five bands, where five, zero or one (mid) band are the most common types (Fig. 1). Classical genetic studies have shown that around nine loci control various aspects of colour and banding patterns in *Cepaea*. Several of these, including the loci responsible for shell ground colour (*C*), band presence/absence (*B*), band interruption *(I*), spread banding (*S*) and band pigmentation (*P*), are in tight linkage on the same chromosome, and are inherited together as a ‘supergene’ (Cain et al. 1960; Cook 1967; Jones et al. 1977; Ramos Gonzalez et al. 2019). Other loci, such as the mid-banded locus (*U*), which controls the suppression of bands 1, 2, 4 and 5, are unlinked.

Genomic analyses promise to advance understanding of colour and patterning loci. In *C. nemoralis*, restriction site-associated DNA sequencing (RAD-Seq) was used previously to map the colour and banding ‘supergene’ with the closest marker ~0.6 centimorgans (cM) from the supergene (Richards et al. 2013). In a subsequent study, zero recombinants were identified between loci within the supergene (Ramos Gonzalez et al. 2019). Now, a draft assembly of the 3.5 Gb *C. nemoralis* genome is available (Saenko et al. 2021), with more than 43,000 predicted protein-coding genes. However, the utility of the assembly for research into the genetics of colour and pattern polymorphism is limited by its contiguity. The high repeat content of the *C. nemoralis* genome (~77%) means that the assembly is made up of more than 28,000 scaffolds, which presents a challenge for genetic mapping studies.

In general, high-density genetic linkage maps are useful to order and orient even fragmented assemblies (Du et al. 2017; Ong et al. 2019; Peng et al. 2020; Tennessen et al. 2017; Varadharajan et al. 2019; Wang et al. 2017), especially when used alongside long-read sequencing and Hi-C capture (Zou et al. 2019). High-resolution linkage maps may also be used to assist in analyses of recombination landscapes and introgression, as well as mapping of Mendelian and quantitative trait loci (Brawand et al. 2014; Bu et al. 2022; Fishman et al. 2001; Guo et al. 2019; Hearn et al. 2022; Hermida et al. 2022; Koch et al. 2021; Ong et al. 2019; Talukder et al. 2019; Lan Zhao et al. 2013). Here, we use Illumina whole-genome resequencing of a laboratory cross to build a linkage map and scaffold the fragmented draft genome assembly of *C. nemoralis* into pseudochromosomes. We then use trait mapping to infer the map position of the shell ground colour (*C*) and mid-banded (*U*) loci, which together represent a major part of the phenotypic variation of the shell.

## Materials and methods

### Whole-genome sequencing of snails from a laboratory cross

We selected a cross previously detailed in Ramos Gonzalez et al. (2019) (cross #11 in Supplementary Table S2). In brief, this was a full-sibling cross, including two parents C451 × C452, 75 offspring that segregated for the shell ground colour (*C*) locus within the supergene, as well as the mid-banded locus (*U*). Two grandparents were also included, making 79 snails in total (Supplementary Table S1).

The parents were C451, pink mid-banded phenotype, genotype *C*^*P*^*C*^*Y*^*U*^*3*^*U*^*-*^, and C452, yellow five-banded *C*^*Y*^*C*^*Y*^*U*^*-*^*U*^*-*^, with the offspring pink mid-banded *C*^*P*^*C*^*Y*^*U*^*3*^*U*^*-*^, pink five-banded *C*^*P*^*C*^*Y*^*U*^*-*^*U*^*-*^, yellow mid-banded *C*^*Y*^*C*^*Y*^*U*^*3*^*U*^*-*^ or yellow five-banded *C*^*Y*^*C*^*Y*^*U*^*-*^*U*^*-*^. Note that this cross also showed segregation for the band pigmentation locus *P* within the supergene, showing no recombinants between the *C* and *P* loci, with an upper confidence limit of 1.76% (Ramos Gonzalez et al. 2019). Zero recombinants between *C*/*B* were reported from other crosses in the same study, with an upper limit of 0.8%. In this study, we focus on the *C* locus, but given zero recombinants within the supergene, the mapping effectively also encompasses the *B* and *P* loci of the supergene because the measured recombination rate is 0%.

The genome of each individual was sequenced at the Wellcome Sanger Institute, using Illumina paired-end methodology (NovaSeq 6000 PE150), aiming for ~10× fold haploid coverage. A single library was prepared from DNA of each individual and run over two lanes. Sequence data were binned by barcode and trimmed for known adapters.

### Linkage map methods

Linkage mapping was performed using Lep-MAP3 (Rastas 2017) because it is able to rapidly produce a map with a large number of markers. First, individual paired-end read files were mapped to the reference genome (GCA_014155875.1) using Bowtie2 v.2.4.1 (Langmead and Salzberg 2012). A VCF file was produced with BCFtools v1.11 (Li 2011) containing the genotype probabilities for all individuals covering the entire genome (~3.5 billion markers). The VCF file was filtered using the following strict criteria to retain only high-confidence SNPs: the quality score was set to the maximum (999), with a minor allele frequency of 0.05; indels were removed, retaining markers where no more than 10% of individuals were missing a genotype, and also retaining markers with an average depth of between 10 and 25 fold.

The filtered dataset included more than two million SNPs. Therefore, a further round of filtering included only SNPs that were heterozygous in parent C451, a snail that was also heterozygous for the shell colour (*C*^*P*^*C*^*Y*^) and mid-banding (*U*^*3*^*U*^*-*^) loci. Additionally, SNPs were excluded where all individuals were heterozygous. The resulting list of SNPs was extracted from the VCF file with vcftools v. 0.1.17 (Danecek et al. 2011), creating a dataset of 314,328 SNP loci across 79 individuals.

The SNP loci were used to call parental genotypes across all contigs with the Lep-MAP3 module *ParentCall2*, with removeNonInformative = 1. The *SeparateChromosomes2* module was run with lodLimit = 9.5 and sizeLimit = 600, and distortionLod = 1 to deal with distorted markers. The *JoinSingles2All* module was run with lodLimit = 5 to assign singular markers to the existing linkage groups (Rastas 2017).

The order of SNPs was determined by running *OrderMarkers2* twenty times, each with six iterations on each of the 22 linkage groups. Markers were phased against the grandparents to ensure no missing data. As others have done (Smith et al. 2020), linkage groups were further refined by evaluating LOD matrices (output using computeLODscores = 1 in OrderMarkers2). SNPs were removed if, after normalisation of LOD scores to values between 0 and 1, the maximum LOD score for any SNP at a location was less than 1 standard deviation from the mean LOD score across all SNPs at the same location (Smith et al. 2020).

SNPs were removed from the map if the contigs they were on were assigned to multiple linkage groups, under the conditions that contigs with 10 or less mapped SNPs were removed from the map completely. The exception was in cases where contigs had >10 SNPs in the map, for which markers were removed only from a single linkage group if the number of SNPs assigned to that LG constituted less than 10% of markers from that contig in the map. Maps were then reconstructed for each linkage group using *OrderMarkers2* (with evaluateOrder and improveOrder = 1 options), with SNPs that failed the filtering criteria set for removal using the removeMarkers option. The order of markers was then evaluated for an additional six iterations.

Lep-MAP3 provides two positions for each marker based on the sex of the parent, male and female. As *C. nemoralis* are hermaphrodites (Cooke et al. 2017), we arbitrarily assigned C451 as the father and C452 as the mother, and the parental positions for each marker were then sex-averaged between the two positions (output with sexAveraged = 1 in OrderMarkers2). Finally, the map was converted to genotypes ‘1 1’, ‘1 2’, ‘2 1’ and ‘2 2’ with the map2genotypes.awk script in Lep-MAP3, where the two digits are inherited from the nominal father, or mother, respectively, for use in the trait mapping analysis (Rastas 2017).

Following the methods in Fishman et al. (2001), the average interval between markers in the linkage map (*s*) was calculated as the sum of the total length of all linkage groups divided by the number of markers minus the number of linkage groups. The expected genome length was calculated by adding 2*s* to each linkage group, to account for terminal chromosome regions, and summing all linkage groups (Fishman et al. 2001). Genome coverage was estimated as the ratio of the cumulative map length in cM to the expected genome size, also in cM (Ren et al. 2016). The genome-wide recombination rate was calculated as the ratio between the cumulative map length and the *C. nemoralis* haploid genome size in Mb (Wilfert et al. 2007).

The genome content completeness of the linkage map as compared to the *C. nemoralis* draft genome was evaluated using BUSCO v5.3.2 (Simão et al. 2015), with the metazoan_odb10 dataset consisting of 954 BUSCOs (Benchmarking Universal Single-copy Orthologs).

### Trait mapping

The complete linkage map with phased genotype data was exported from Lep-MAP3, and the genotypes were converted to alleles matching our sample population as a F2 cross. An in-house R script was used to convert genotypes following the convention of ‘1 2’ and ‘2 1’ = ‘AB’; ‘1 1’ = ‘AA’, and ‘2 2’ = ‘BB’. Binary trait mapping of shell ground colour and mid-banded phenotype used the *qtl2* package implemented in R (Broman et al. 2003, 2019). *qtl2* is capable of handling high-density mapping and genotype data, and allows for the association mapping of binary traits through logistic regression analysis (Broman et al. 2019).

To begin, the module *calc_genoprob()* was utilised to calculate genotype probabilities with a hidden Markov model, with recommended settings and assuming a genotyping error probability of 0.002. Then, a genome scan was performed to evaluate the association between the genotype and each phenotype (yellow/not yellow and mid-banded/not mid-banded) using logistic regression analysis for binary traits (module *scan1* with model = binary). This produced an LOD score, where the null hypothesis was that there were no loci anywhere in the genome associated with the binary trait, and the alternate hypothesis was that there was a binary trait locus near a specific position. The LOD scores were plotted across the linkage groups to identify peaks associated with a putative binary trait locus.

The threshold of the significance level of the LOD score was determined by a permutation test for each binary trait with the recommended number of permutations (module *scan1perm*, model = binary, n_perm = 1000) (Broman et al. 2019). All binary trait loci with an LOD score greater than the calculated threshold at 95% (*P* = 0.05) were considered significant. Utilising a Bayesian credible interval, these thresholds were then used to identify LOD peaks on the genomic map associated with each of the phenotypes, either yellow or mid-banded.

Multiple peaks across a chromosome were identified using the module *find_peaks* (threshold = (*P*), peakdrop = 1.8), where LOD peaks above the significance level threshold were considered independent only if the LOD score dropped by at least 1.8 between the lower limits of the two adjacent peaks. The effects of the different genotypes on the phenotypic traits, so-called allele effects, were estimated for individual linkage groups (module *scan1coef*, model = binary); this indicates the likely position of the binary trait locus, and ascertains which genotypes are associated with the presence/absence of the phenotype in question. Finally, putative locations for the colour/mid-banding locus on the linkage map were examined for each phenotype against the raw, phased genotypes.

## Results

### Linkage map construction

The final linkage map was visualised using the *LinkageMapView* package implemented in R (Ouellette et al. 2018) and is shown as a full linkage map (Supplementary Fig. S1) and as a density map (Fig. 1).

Of 314,328 SNPs in the filtered VCF file, a total of 215,593 were used in the final linkage map (Supplementary Table S2), assigned to 22 linkage groups (Table 1 and Supplementary Table S3). The cumulative length of all linkage groups in the map is 2143 cM, with an average interval between loci of 0.0099 cM. The number of markers per linkage group varied from 1367 (LG22) to 59,069 (LG1), with an average of 9800 per group. The linkage groups varied in size from 56.84 cM on LG22 to 151.41 cM on LG1 (Supplementary Table S3). The estimated full-length genome was 2187 cM, giving a genome coverage of ~98%, implying that most recombination events were identified. There was considerable variation in marker density between linkage groups, ranging from one SNP per 0.0025 cM (LG1) to one per 0.423 cM (LG22) (Fig. 1). The genome-wide recombination rate was 0.61 cM/Mb.

**Table 1:** Summary statistics for each *Cepaea nemoralis* linkage group.

Linkage group	No. linked SNPs	Total size of contigs (Mb)	Linkage length (cM)	Per cent of linkage map
1	59,069	265.37	151.48	7.07
2	10,648	54.22	92.54	4.32
3	16,584	105.28	104.53	4.88
4	9842	52.78	136.18	6.35
5	9096	52.12	70.11	3.27
6	12,030	73.1	134.52	6.28
7	6101	38.83	84.33	3.93
8	10,653	70.53	126.84	5.92
9	6495	39.32	81.74	3.81
10	9104	45.96	89.21	4.16
11	5746	38.69	74.47	3.47
12	5197	36.91	109.44	5.11
13	7352	45.71	87.46	4.08
14	8217	58.09	104.52	4.88
15	4387	49.68	110.05	5.13
16	5025	22.32	60.85	2.84
17	3683	39.84	90.25	4.21
18	5173	39.18	93.32	4.35
19	10,104	59.01	96.44	4.50
20	7000	45.86	116.55	5.44
21	2720	16.74	72.04	3.36
22	1367	13.34	56.84	2.65
Total	215,593	1262	2143	100
Mean	9799	57	97	

The linkage map includes 6445 contigs, comprising ~1.21 Gb or 34.59% of the *C. nemoralis* genome by size but representing 22.58% of the contigs in the genome. The contigs placed in the map are larger, with a greater mean and median size (187,381 bp and 90,722 bp, respectively) than the contigs not placed in the map (103,348 bp and 33,960 bp mean and median, respectively) (Supplementary Fig. S2). The majority of contigs not included in the map were filtered out by the software prior to map construction due to a lack of parentally informative markers (Rastas 2017).

We assessed the genome content completeness of the contigs included in the linkage map with BUSCO v5.3.2 (Simão et al. 2015) and compared the results to the full *C. nemoralis* draft genome (Saenko et al. 2021). Of the 954 metazoan BUSCOs, 401 (42%) were identified in the contigs included in the linkage map as complete (362, or 37.9% as single copy, and 39, or 4.1% as duplicated) compared to 87.2% (709, or 74.3% and 123, or 12.9 % as single copy and duplicated genes, respectively) for the *C. nemoralis* genome (Saenko et al. 2021). The linkage map, therefore, includes around half (48%) of the complete BUSCOs identified in the draft genome, while representing ~35% of the full assembly by size. A further 36 genes (3.8%) were identified as fragmented, with 512 (54.2%) missing compared to 36 (3.8% fragmented and 89 (9%) missing in the complete assembly.

### Trait mapping

**Fig. 2:**
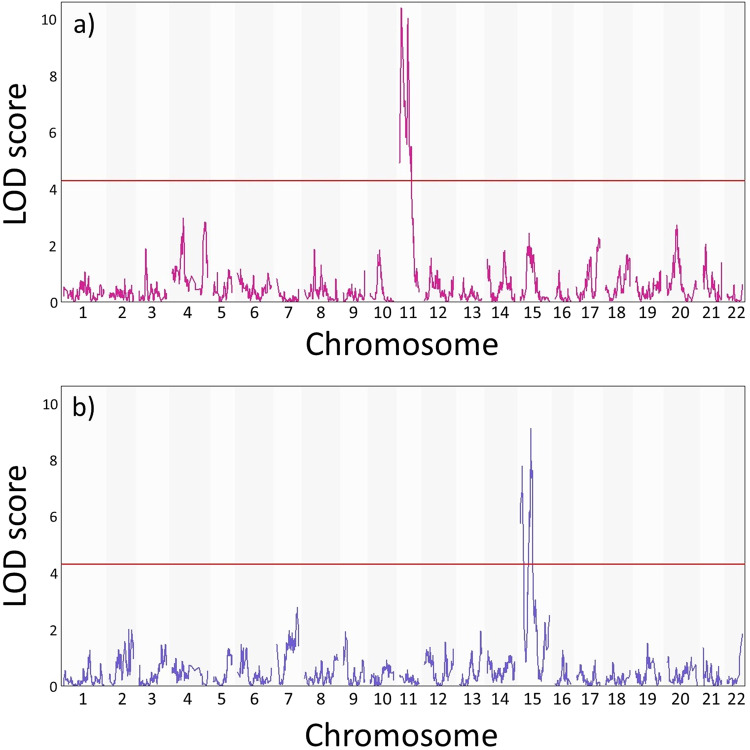
Results of the genome scans showing LOD scores across all linkage groups. **a** Results for the yellow phenotype and **b** results for the mid-banded phenotype. The red line indicates the 5% genome-wide significance level derived from the permutation test.

We investigated associations between genome position and the binary trait of presence (1) or absence (0) of a yellow shell, the trait described by shell ground colour locus *C*, using logistic regression with a genome scan to identify peaks in LOD scores across each chromosome (Fig. 2a). The analysis showed a pronounced peak on linkage group 11 for the yellow phenotype, with an LOD threshold of 4.28 at 5% significance level. There were two separate Bayesian credible intervals on the linkage group that had LOD scores higher than the significance threshold; the first is found between positions 6.757–10.878 cM (interval Y1) and the second from position 31.385–33.398 cM (interval Y2), made up of three and four map positions, respectively (Table 2).

**Table 2:** LOD peaks for yellow and mid-banded phenotypes.

Phenotypic trait	Interval ID	CHR	Position	LOD score	Start position	End position
Yellow colour	Y1	11	7.429	10.409	6.757	10.878
Yellow colour	Y2	11	32.727	10.042	31.385	33.398
Mid-banded	M1	15	6.832	7.789	4.092	8.175
Mid-banded	M2	15	40.182	9.12	38.84	44.218

**Fig. 3:**
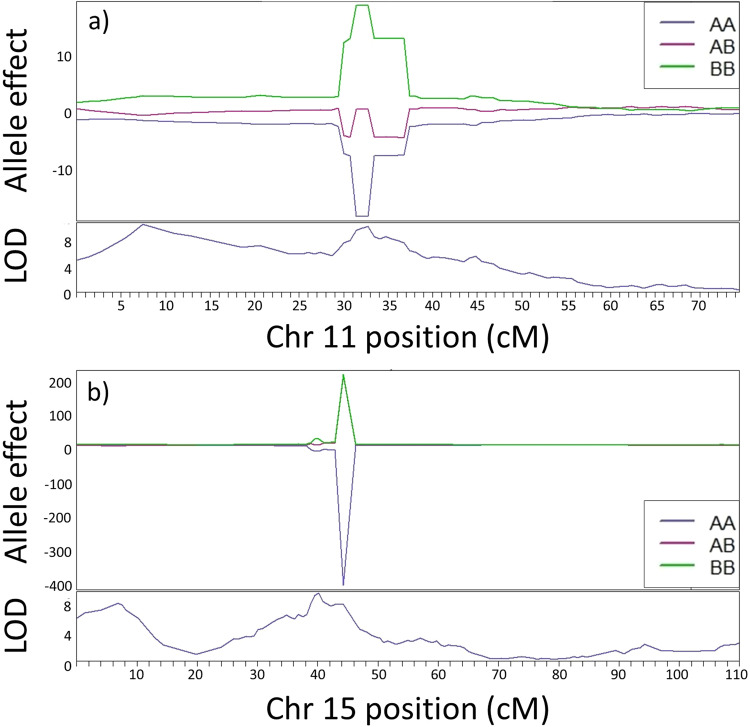
Effect plots of genotypes for each phenotype investigated with associated LOD scores. **a** Effects of the different genotypes on the presence of yellow shell colour phenotype on chromosome 11. **b** Effects of the different genotypes on the presence of the mid-banded phenotype on chromosome 15.

The phenotype of each individual was compared against the raw, phased genotypes along linkage group 11 (Fig. 3a). As expected, one genotype (‘BB’) had a positive effect on yellow phenotype, while the other (‘AA’) had a negative effect on yellow phenotype, with genotype ‘AB’ falling between the two. However, while the largest peak in LOD score was around position ~7.4 cM, within interval Y1 in Table 2, there was no corresponding peak in allele effect (Fig. 3a). In comparison, the other peak in LOD score at around 32.7 cM, interval Y2 in Table 2, also had a corresponding peak in allele effect (Fig. 3a).

**Fig. 4:**
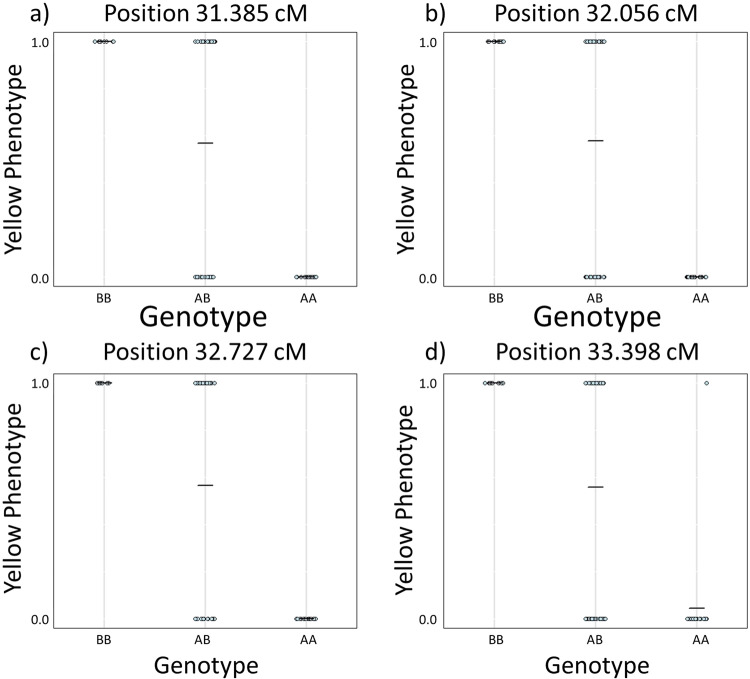
Raw genotypes for all individuals at each putative locus associated with yellow shell ground colour within the interval Y2 (1 = yellow, 0 = not yellow). **a** Raw genotypes at position 31.385, **b** raw genotypes at position 32.056, **c** raw genotypes at position 32.727, **d** raw genotypes at position 33.398. All positions except one, 33.398 **d**, show the same segregation pattern between alleles, where allele B is associated with yellow shell ground colour and A is associated with a non-yellow.

In the first interval (Y1), three map locations (Supplementary Fig. S3) were defined by 8 SNPs. However, despite this interval exhibiting a LOD score well above the significance threshold, these positions did not conform to the expected allele pattern for the yellow phenotype, because there were two yellow individuals with an ‘AA’ genotype. Since the A allele is associated with ‘not yellow’, we ruled out these locations as containing the colour locus. In comparison, of the four map locations in the second interval (Y2), three positions (31.385, 32.056 and 32.727) perfectly matched the expected allele pattern (Fig. 4) where all BB individuals have a yellow phenotype, all AA individuals have a not yellow (i.e., pink) phenotype and AB individuals are split between the two traits.

Therefore, the most likely candidate position for the location of the shell ground colour locus *C* is a ~1.3 cM region around positions 31.385, 32.056 and 32.727, defined by 32, 155 and 305 SNPs, respectively. All individuals conform to the expected allele pattern for the phenotype within this region. These three positions are represented by nine contigs (Supplementary Table S4), with two contigs shared across positions 31.385 and 32.056, and one shared between 31.385 and 32.727.

The same methods were used to map the mid-banded locus *U*. The genome scan showed a marked peak in LOD scores on linkage group 15, with an LOD threshold of 4.29 at 5% significance level (Fig. 2b). Again, two separate Bayesian credible intervals with LOD peaks above the threshold were identified (Table 2). The first interval (M1) was between positions 4.092 and 8.175 cM (peak ~6.8 cM; interval M1), with the second higher peak between positions 38.840 and 44.218 cM (peak 40.2 cM; interval M2, Table 2). Despite exhibiting a LOD score above the significance threshold, none of the positions within this interval exhibited allele effects alongside the LOD score (Fig. 3b), whereas the second interval showed a pronounced peak in allele effect, with the ‘BB’ genotypes having a positive effect on mid-banded phenotype (i.e., BB is positively correlated with mid-bandedness), and AA having a negative effect on mid-banded phenotype.

**Fig. 5:**
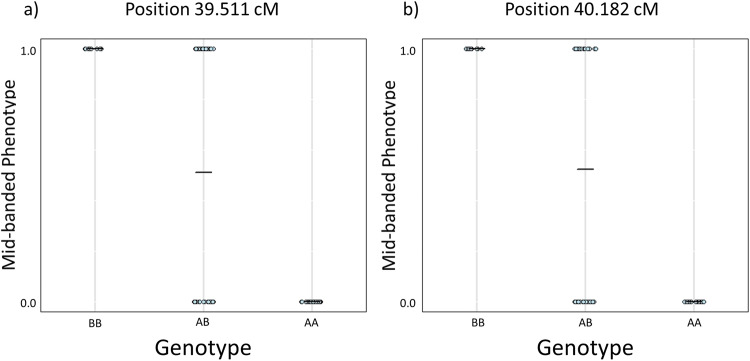
Raw genotypes for all individuals at each putative locus associated with the mid-band phenotype within interval M2 (1 = mid-banded, 0 = not mid-banded). **a** Raw genotypes at position 39.511 and **b** showing raw genotypes at position 40.182. The two positions shown here are the only two positions within this that fit the expected pattern, such that all BB genotypes correspond to mid-banded phenotype, and all AA genotypes correspond to lack of a mid-banded (usually five-banded). The other six locations within this interval did not fit the pattern and are shown in Supplementary Fig. S5.

In the first interval M1, the three map locations (Supplementary Fig. S4) were defined by 21 SNPs. However, none of the three map positions showed the expected allele pattern for the mid-banded phenotype. In comparison, two map positions out of eight in interval M2 followed the expected pattern, at positions ~39.511 and ~40.182 (Fig. 5 and Supplementary Fig. S5). All mid-banded individuals exhibited a BB genotype, and all non-mid-banded individuals had an AA genotype, with individuals with an AB genotype split across the two phenotypes.

Therefore, the most likely candidate positions for the location of the mid-banded locus *U* is a ~0.7 cM region around positions 39.511 and 40.182 cM on linkage group 15, because all individuals conform to the expected allele pattern for the phenotype within this region. These two positions are represented by 398 SNPs across 21 contigs, with eight shared across the two positions (Supplementary Table S5).

## Discussion

We generated the first high-density linkage map for the land snail *Cepaea nemoralis*. Trait mapping was then used to identify two separate chromosomal regions that contain the shell ground colour *C* and mid-banded loci *U*. As we have previously not detected any recombination events within the supergene region (Ramos Gonzalez et al. 2019), then the region that contains *C* also likely contains the other main component loci of the supergene, including banding *B* and pigmentation *P*. The work adds value to the existing genome assembly by establishing the physical position between previously unmapped scaffolds. The findings also establish a baseline from which to identify candidate genes for phenotypic traits, such as the shell ground colour and banding loci.

Previous works on the functional molecular genetics of the shell colour of *C. nemoralis* have not yielded definitive answers concerning the key players. Mann et al. (2014) deployed proteomic analysis of mantle tissue to identify several proteins involved in shell production but did not find any candidates directly associated with shell pigmentation. Kerkvliet et al. (2017) used differential expression and analysis of SNPs to identify candidate genes involved in shell pigmentation, and suggested that genes inhibiting the production of melanin in melanocytes could be related to the bands on the shell. More recent work has ruled out melanin as playing a role in either shell or banding pigmentation (Affenzeller et al. 2020). Our view is that the genetic regulatory networks and key controlling loci must be identified to then exploit the full potential of these functional ‘omic studies.

### Linkage map

The map was constructed using more than 6000 contigs and ~215,000 SNPs, which include about 35% of the bases of the *Cepaea nemoralis* genome of ~3.5 Gb and about half of the expected BUSCO genes. The mapped contigs represent only ~23% of all the contigs in the *C. nemoralis* assembly (Saenko et al. 2021) because they are disproportionately large relative to the contigs that were not included (Supplementary Fig. S2). As gastropod genomes, in general, tend to have a high repeat content, estimated at 77% in *C. nemoralis* (Saenko et al. 2021), the linkage map provides a good representation of the non-repetitive, gene-rich regions of the *C. nemoralis* genome. Unmapped contigs and scaffolds are more likely to be repetitive and less likely to contain functionally important genes.

The results suggest an average genome-wide recombination rate of ~0.61 cM per Mb. This rate is low but within the observed range for invertebrates (Wilfert et al. 2007) and not substantially different from the recombination rate reported for another gastropod snail, *Biomphalaria glabrata* (0.8 cM per Mb; Tennessen et al. 2017), although this particular snail has also been reported with a substantially larger recombination rate of 1.9 cM per Mb (Bu et al. 2022). Of course, the rate will be an underestimate, albeit slight, if recombination events outside the linkage groups are missed. It is also worth emphasising that there is wide variation in recombination rate between regions and chromosomes.

*C. nemoralis* has 22 chromosome pairs (Gill and Cain 1980; Page 1978). Similarly, the linkage map assigned contigs into 22 linkage groups, with LG1 substantially larger than the other linkage groups, accounting for ~27% of all markers in the linkage map, and ~24% of the mapped genome by size. There was wide variation in the relationship between recombination and the number of SNPs in each group. For example, LG1 has ~3.5 times the number of SNPs (27% of the total) when compared with the second most SNP-rich linkage group, LG3, while being only ~1.5 times larger in cM (Supplementary Table S3). The cumulative map length in cM of all linkage groups in the map (~2100 cM) is larger than maps reported for other gastropods, such as that of *B. glabrata* (~700 cM; Tennessen et al. 2017; ~1700 cM; Bu et al. 2022), *Littorina saxatilis* (~1100; Koch et al. 2021; Westram et al. 2018) and that of *Cerastoderma edule* (1073 cM; Hermida et al. 2022). One part of the explanation for the difference may be that the *Cepaea* linkage map contains a much larger number of markers than the above maps (~49,000; Tennessen et al. 2017 and 996 SNPs; Bu et al. 2022 in *B. glabrata*, ~19,000 SNPs for *Littorina saxatalis*; Koch et al. 2021 and ~13,000 SNPs for *C. edule*; Hermida et al. 2022).

The finding of a single large linkage group is consistent with karyotyping (Gill and Cain 1980; Page 1978; Richards et al. 2013). Similar karyotypes with one very large chromosome have been reported in other snails in the Helicidae, such as *Eobania vermiculata* and *Otala lactea* (Petraccioli et al. 2021). The number of chromosome pairs in *C. nemoralis* (*n* = 22) is on the low side compared with others in the superfamily Helicoidea, which range from *n* = 22 to *n* = 30 (Petraccioli et al. 2021), so perhaps the largest pair is derived from one or more fusion events between chromosomes (Petraccioli et al. 2021)? In other groups of animals, inferences of ancestral linkage groups have revealed deep patterns of chromosomal organisation that are only accessible with high-quality linkage maps and reference genomes (Damas et al. 2022; Farré et al. 2016). Tennessen et al. (2017) have made progress in understanding chromosome evolution in gastropod snails, and as further linkage maps and whole chromosome assemblies become available, understanding chromosomal rearrangements and synteny within the stylommatophoran group of snails will be a fruitful field of study, especially in understanding the repeated evolution of shell polymorphism, and the formation of supergenes.

Most markers not included in the map were filtered out during the first step of the linkage mapping analysis, as these markers were deemed uninformative. This could be due to low coverage across individuals or because the sib-parents were not heterozygous at any of the markers called and thus not recombination informative (Rastas 2017). The former is perhaps more likely, as our dataset was specifically filtered prior to linkage mapping to include only SNPs that were heterozygous in the pink parent; thus, all markers available for the linkage mapping software were theoretically informative for linkage analysis. Another explanation might be that the reads were misaligned due to paralogous sequences in the genome, because of the high repeat content in *C. nemoralis*. Future improvements may incorporate markers that do not follow this pattern to allow for a broader approach to phenotype/genotype association analysis and aim to include contigs not currently on the map. Nonetheless, despite these considerations, we are confident that the linkage map is an important resource to add to the draft genome of the assembly of *C. nemoralis*.

### Mapping *Cepaea nemoralis* phenotypes

Binary trait mapping was used to map both the shell ground colour locus *C* and the mid-banded locus *U*. The most likely position for *C* is in a ~1.3 cM region around 31.385–32.727 cM on linkage group 11, represented by 492 SNPs and nine contigs (Supplementary Table S4). Likewise, the most likely candidate position for *U* is a ~0.7 cM region around 39.511 and 40.182 cM on linkage group 15, represented by 3 SNPs across 21 contigs. These findings are concordant with previous inferences using classical genetics that showed that the mid-banded locus *U* is unlinked to the supergene that includes *C* (Cain et al. 1960; Cook 1967).

Both analyses of shell ground colour and banding initially revealed two separate credible regions within a single linkage group, 11 and 15, respectively. One explanation is that either the linkage map and/or the reference genome have assembly errors. The association may also be due to an effect of the population structure within the dataset functioning as a confounding factor and causing false positives due to the kinship between our inbred samples. Stratification within a population can result in spurious associations in a number of genetic studies including genome-wide association and case/control studies, if not corrected (Campbell et al. 2005; Tian et al. 2008), and this becomes more challenging when family structure, or kinship, is also present (Price et al. 2010). Unfortunately, kinship is an effect that currently cannot be accounted for when mapping binary traits (Broman et al. 2019; McClatchy et al. 2018). Alternatively, the association could be a random effect in the data caused by the decline in association between genotype and phenotype across the chromosome. Whichever the explanation, only one of the regions showed a corresponding phenotypic effect, meaning that at this location, the genotypes likely do not control the phenotype in question, and therefore do not contain the loci in question.

A further potential limitation of the study is the relatively small sample size of 75 F2 individuals. In a similar manner to Bu et al. (2022), we used a power analysis (Hu and Xu 2008) to estimate the statistical power to detect trait loci, assuming a conservative scenario of the 2 and 5 cM confidence intervals identified for the yellow and mid-banded phenotypes (R function available; see Johansen 2023). Using a trait variance of 100% (both shell ground colour and mid-banding are entirely genetically determined, and type I error rate of *α* = 0.01, the power to detect trait loci would be 100% for both intervals. The trait variance would have to be below ~40% before the power reduces to less than 100% (for 5 cM interval), and ~21% for the power to be less than 95%.

Despite these considerations, an improved assembly and/or linkage map is a necessity in any future trait mapping in *Cepaea*. The map regions identified are sequence-incomplete, both because of the draft nature of the assembly and because of the rigorous filtering during processing. A further consideration is that there are likely to be assembly and other errors in the existing contigs, such as false SNPs caused by repetitive paralogous sequences, which did not segregate properly in the map. For example, it is otherwise difficult to explain why SNPs from a contig such as tig00045252 (Supplementary Table S4) are found at map positions 31.385, 32.056, 32.727 and 33.398, yet the same positions also coincide with multiple other contigs.

### Genetics of shell characteristics—a new start

Here we have presented the first high-density, complete linkage map for *Cepaea nemoralis* and mapped two important shell phenotype loci. The shell ground colour locus *C* identifies a limited region of linkage group 11 within which the colour and pattern supergene must reside. For the first time we show that the locus *U* controlling for the mid-banded phenotype is on an unlinked region in linkage group 15. We look forward to the further integration of the linkage map with improved *C. nemoralis* genome assemblies to fully define the supergene region and this accelerated discovery and understanding of the causative loci. It will be particularly fruitful to compare genes expressed in the mantle with candidates identified by linkage mapping, and to better define the gene regulatory networks controlling trait expression. The linkage map will also be useful in studies of synteny across related species and as a resource in population genomic studies of *C. nemoralis*.

In their famous paper, Jones et al. (1977) questioned the possibility of understanding polymorphism in *Cepaea* as possibly ‘a problem with too many solutions’. We hope that the whole-genome linkage map presented here, as well as the mapping of shell-polymorphism loci can serve as a further impetus to understand the genetics underlying the polymorphism in *Cepaea*, as well as the much wider group of colour polymorphic snails.

### Supplementary information

Supplementary Tables S4 and S5 and Supplementary Figures S2–S5

Figure S1

Table S1

Table S3

Table S2

## Data Availability

The raw sequence reads are available under BioProject accession PRJEB36910 on the International Nucleotide Sequence Database Collaboration (INSDC https://www.insdc.org/). The script used for the power analysis is available at https://github.com/M-Johansen/QTL_power_calculation. All relevant processed data are in the tables and Supplementary Material.
