# Supplementary material for: Fine mapping of the Cepaea nemoralis shell colour and mid-banded loci using a high-density linkage map
Source: Heredity (Edinb). 2023 Sep 27;131(5-6):327–37. doi: 10.1038/s41437-023-00648-z (PMC10673960; doi:10.1038/s41437-023-00648-z)
Supplement: Supplementary file 1 — Supplementary Tables S4 and S5 and Supplementary Figures S2–S5 [file 41437_2023_648_MOESM1_ESM.pdf]

Table S4. Overview of map positions within interval Y2, conforming to expected allele pattern for yellow phenotype. Two contigs are shared across the first and second position (tig00008644 and tig00045252), while one is shared between all three positions (tig00045252).

Position (cM)	Contig	No. SNPs
31.385	ctg23821	9
	ctg31541	21
	ctg32869	1
	ctg56065	1
	tig00008644	20
	tig00045252	3
	tig00094642	55
	tig02169229	3
	tig02170041	74
32.056	tig00045252	218
	tig00008644	78
32.727	tig00045252	9

Table S5. Overview of the two map positions conforming to expected allele pattern for mid-banded phenotype in interval M2. Eight contigs (tig00399928, tig00395755, contig_38459, ctg13129, ctg24871, ctg10236, tig00048729 and contig_29810) are shared across both positions.

Position (cM)	Contig	No. SNPs
**39.511**	tig00399928	54
	tig00395755	29
	contig_38459	17
	ctg13129	15
	ctg24871	15
	ctg10236	11
	ctg32510	7
	tig00048729	6
	contig_29810	4
	ctg14581	2
	ctg39949	1
**40.182**	contig_38459	88
	tig00399928	46
	ctg13129	20
	tig00395755	19
	ctg9975	15
	ctg4665	9
	contig_29810	8
	ctg24871	5
	ctg10236	4
	ctg11370	4
	ctg15786	4
	tig00048729	3
	ctg12323	3
	ctg27906	3
	tig02174880	3
	contig_23386	1
	ctg11237	1
	ctg43997	1

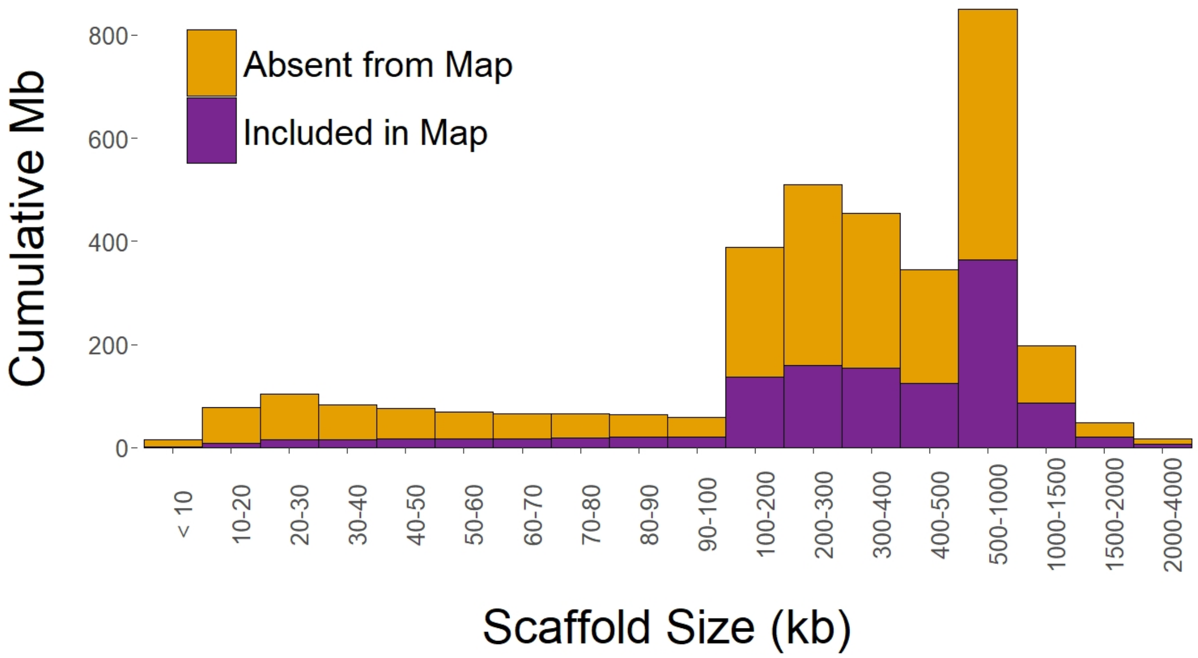

Figure S2: Comparison of the sizes of contigs from the *C.nemoralis* genome that were/were not included in the final linkage map.

Figure S3: Raw genotypes for all individuals, at all SNPs at each putative locus within yellow interval Y1, 1=yellow. None of these positions conform to the segregation pattern we expect to see between alleles for yellow phenotypes

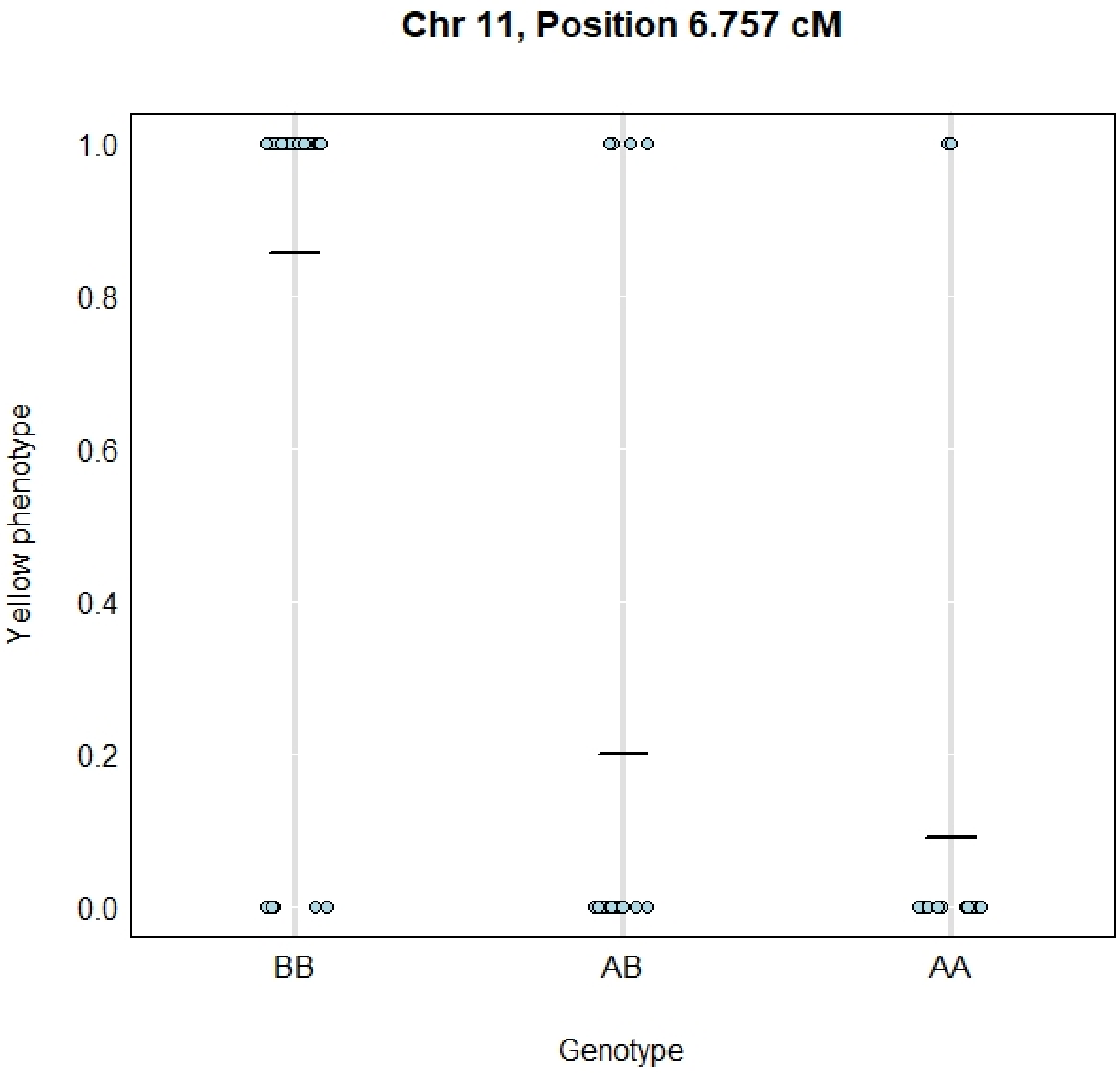

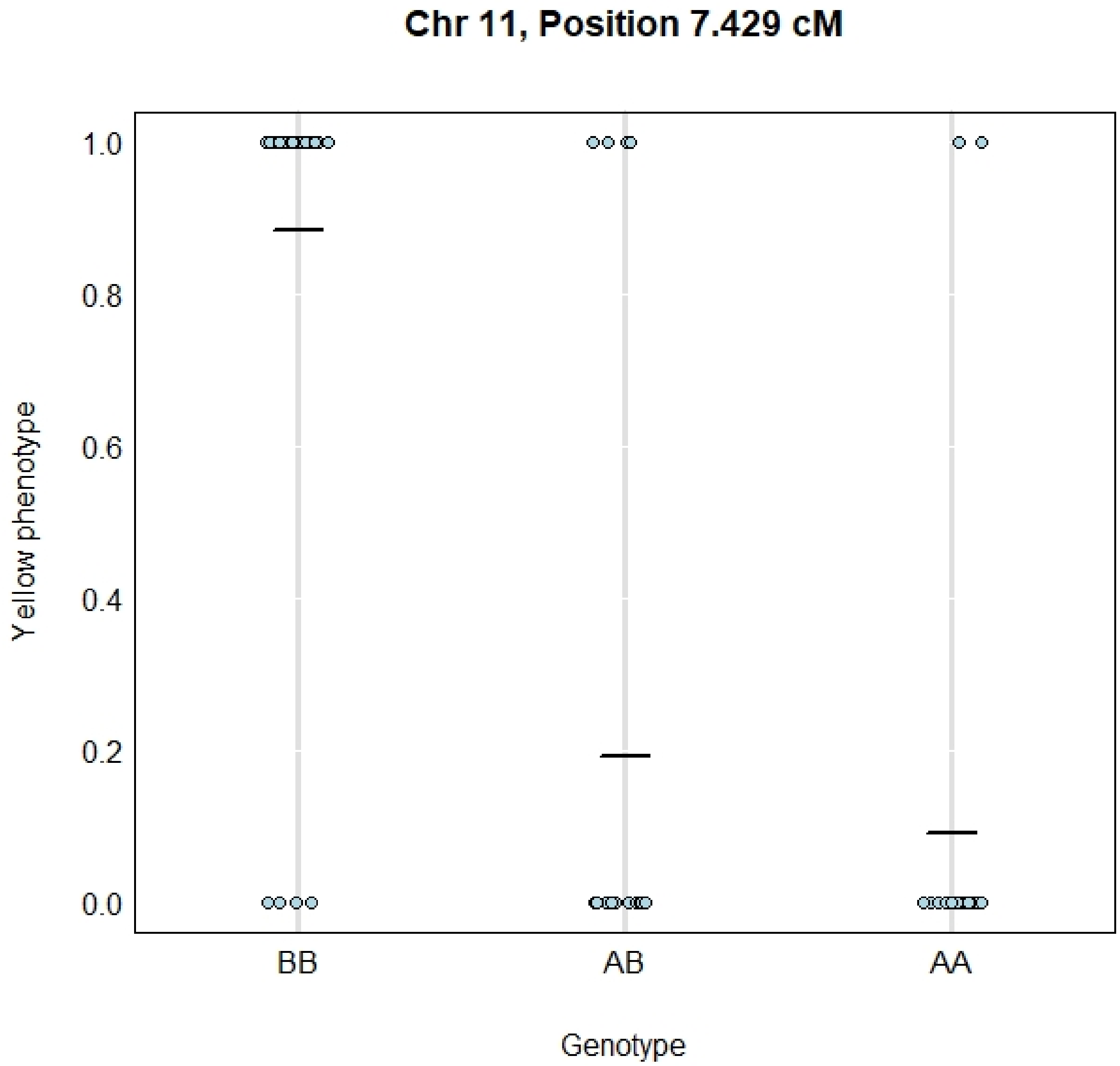
Chr 11, Position 7.429 cM
Yellow phenotype
1.0
0.8
0.6
0.4
0.2
0.0
BB
AB
AA
Genotype

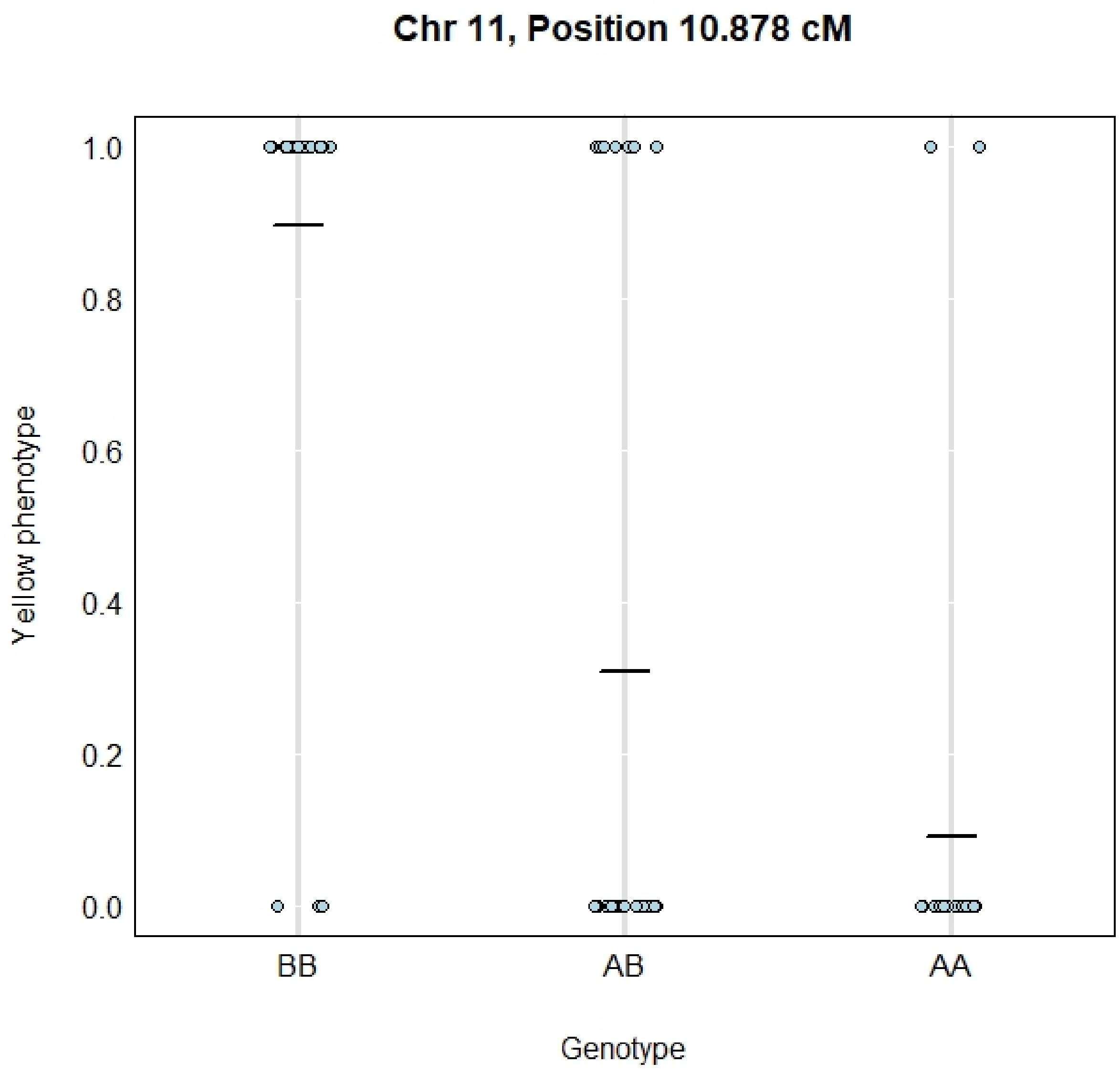
Chr 11, Position 10.878 cM
Yellow phenotype
1.0
0.8
0.6
0.4
0.2
0.0
BB
AB
AA
Genotype

Figure S4: Raw genotypes for all individuals, at all SNPs at each putative locus within mid-banded interval M1, 1=mid-banded. None of these positions conform to the segregation pattern we expect to see between alleles for yellow phenotypes.

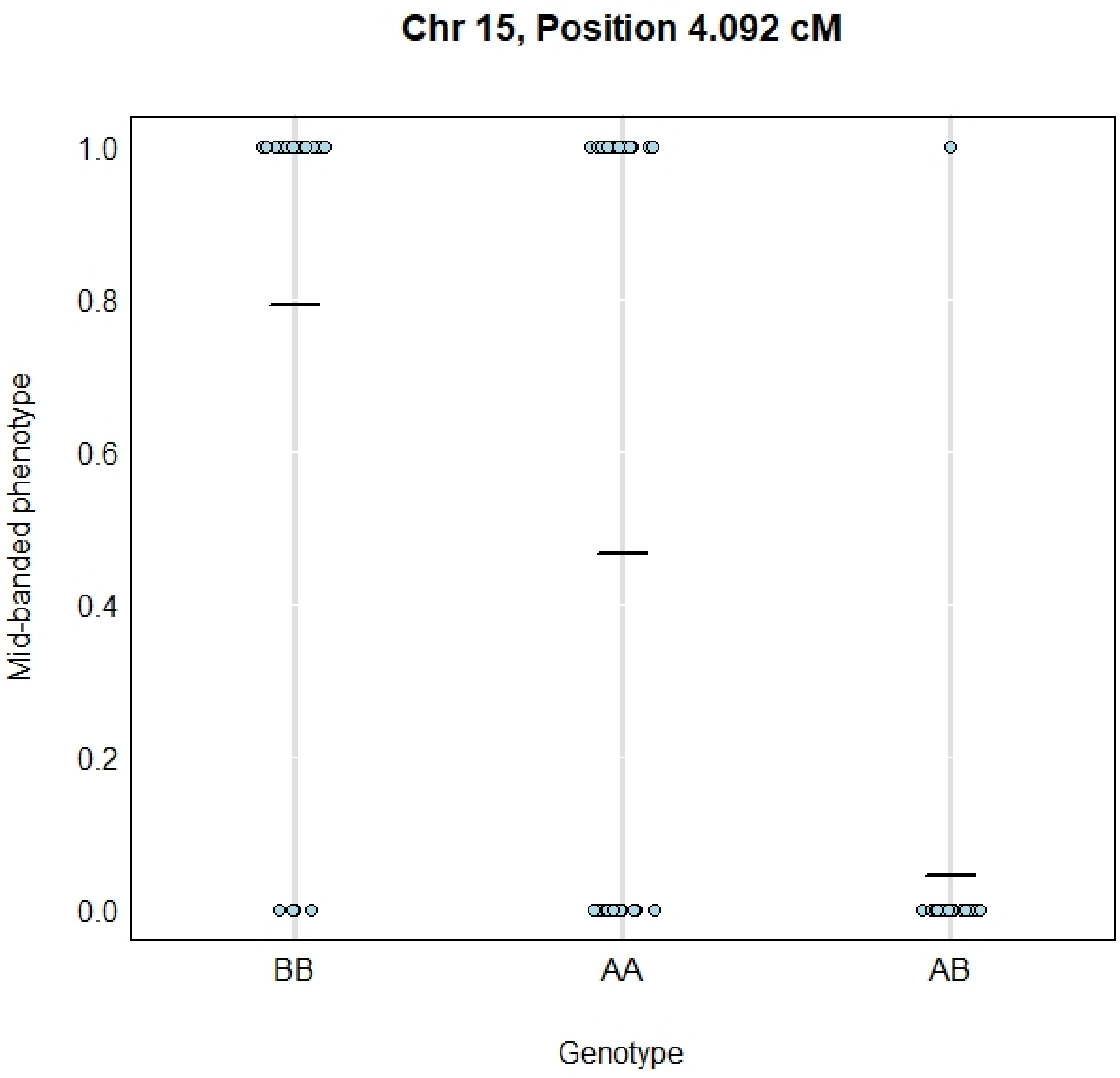

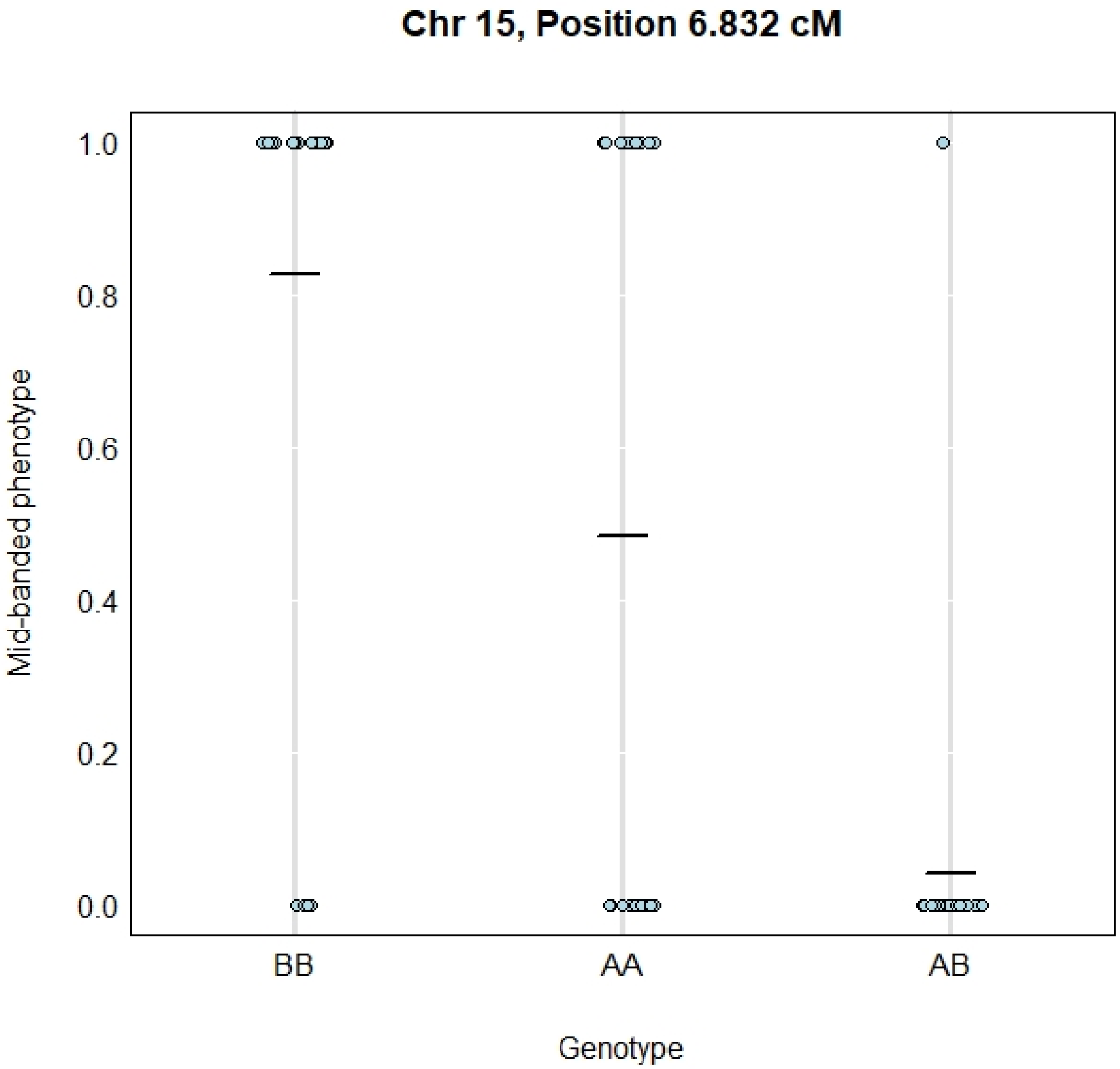
Chr 15, Position 6.832 cM
1.0
0.8
0.6
0.4
0.2
0.0
Mid-banded phenotype
BB
AA
AB
Genotype

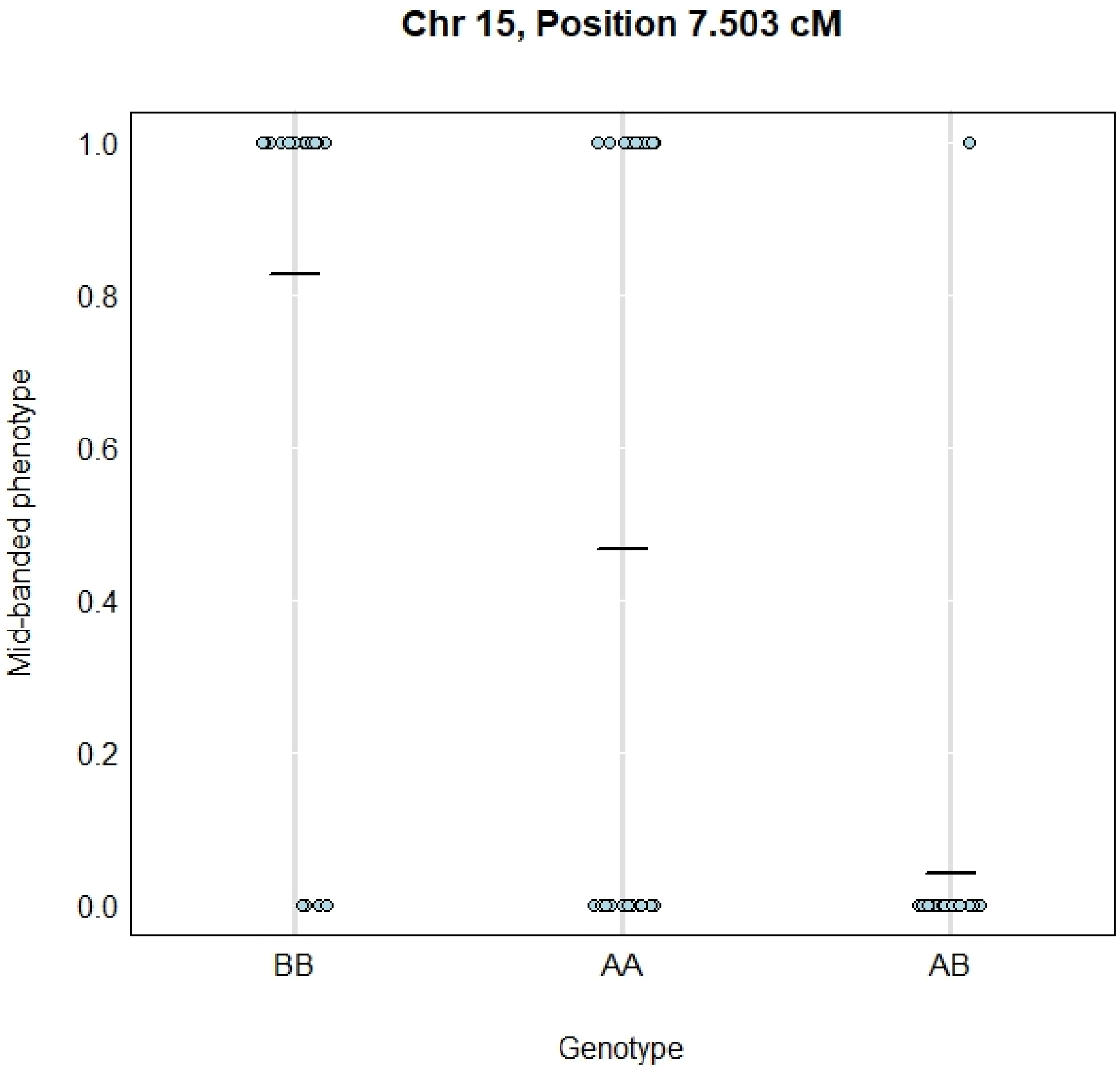
Chr 15, Position 7.503 cM
1.0
0.8
0.6
0.4
0.2
0.0
Mid-banded phenotype
BB
AA
AB
Genotype

Figure S5: Raw genotypes for all individuals, at all SNPs at each putative locus within mid-banded interval M2 that do not fit the expected allele pattern.

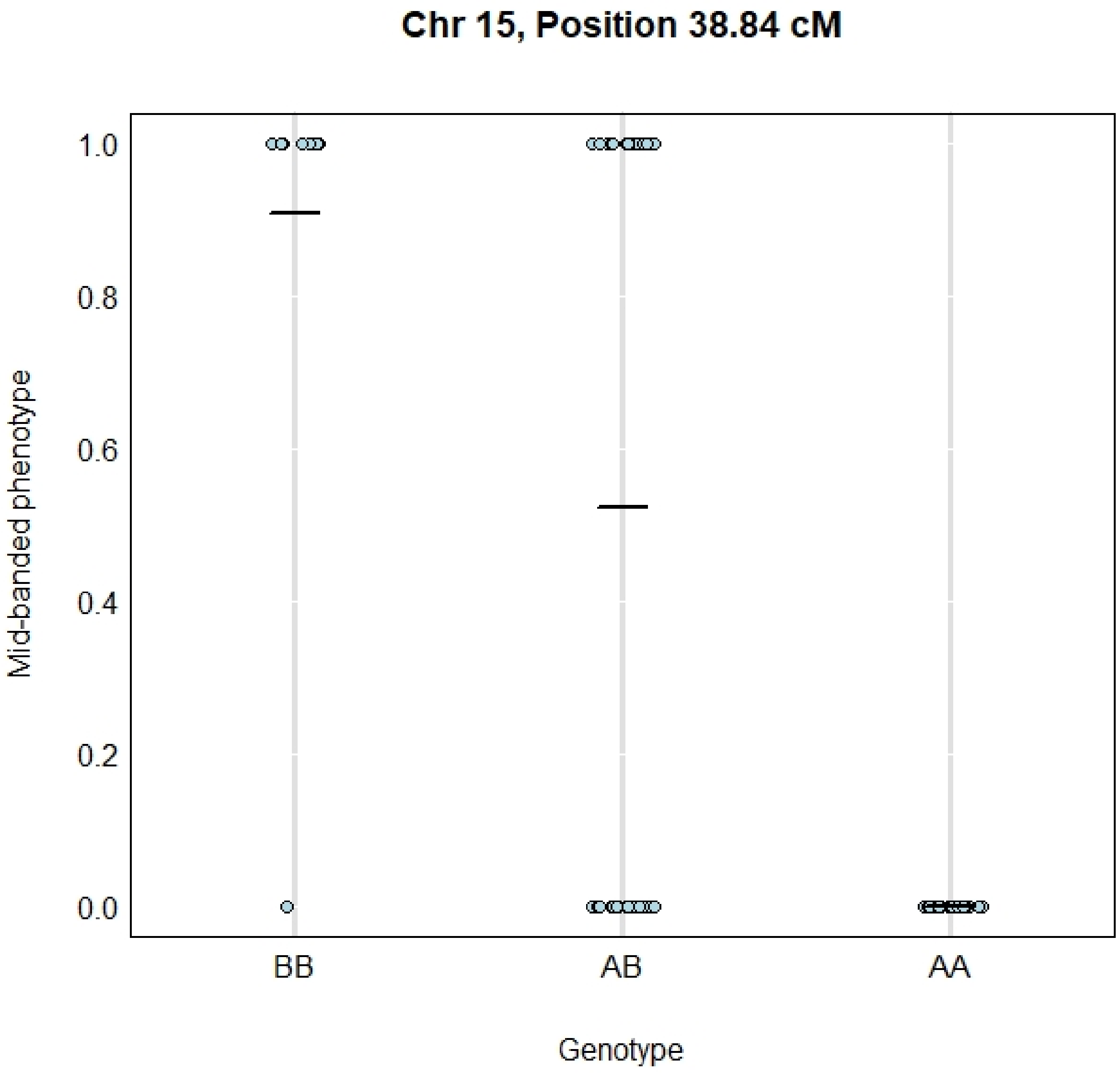

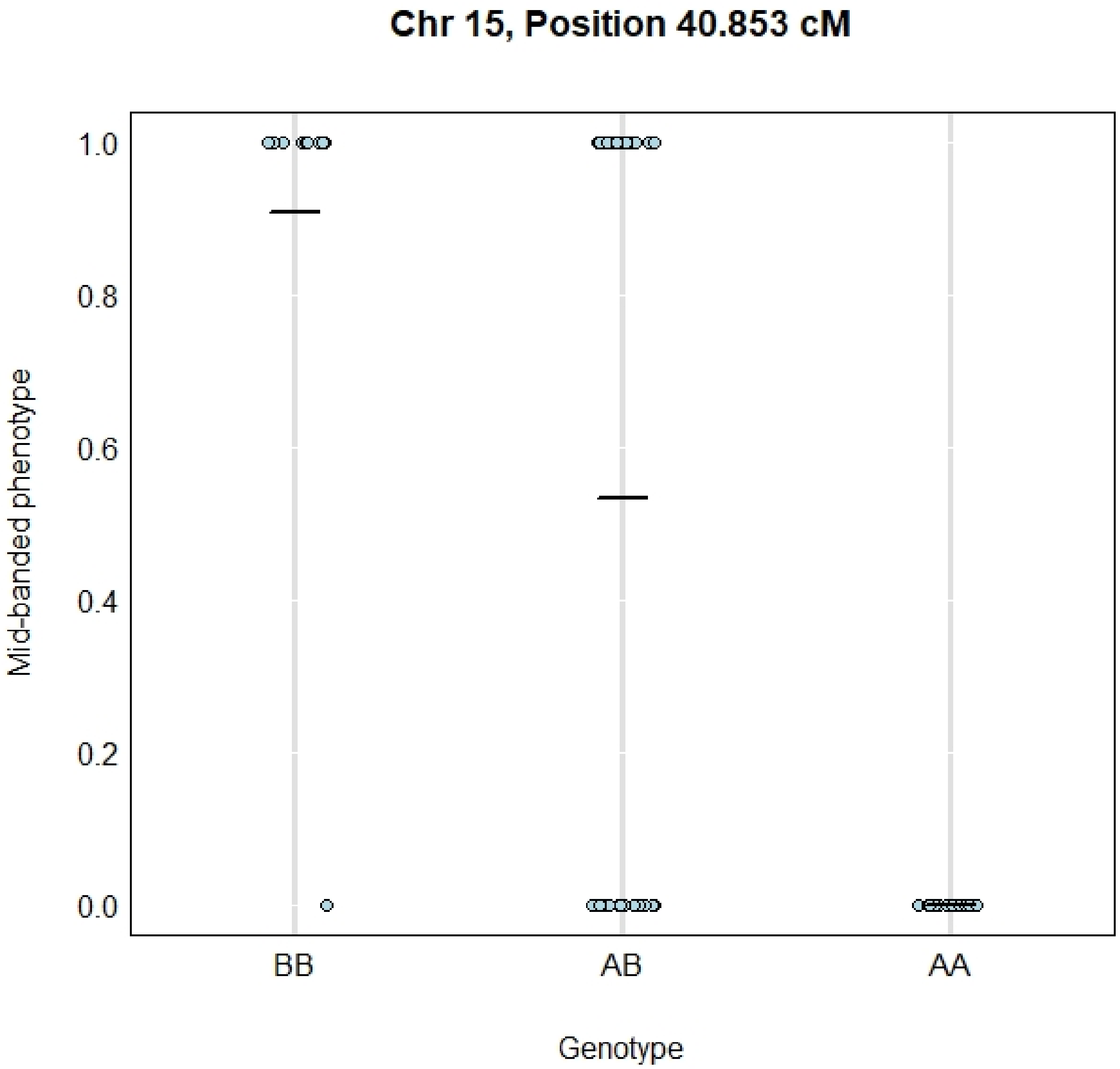
Chr 15, Position 40.853 cM
Mid-banded phenotype
1.0
0.8
0.6
0.4
0.2
0.0
BB
AB
AA
Genotype

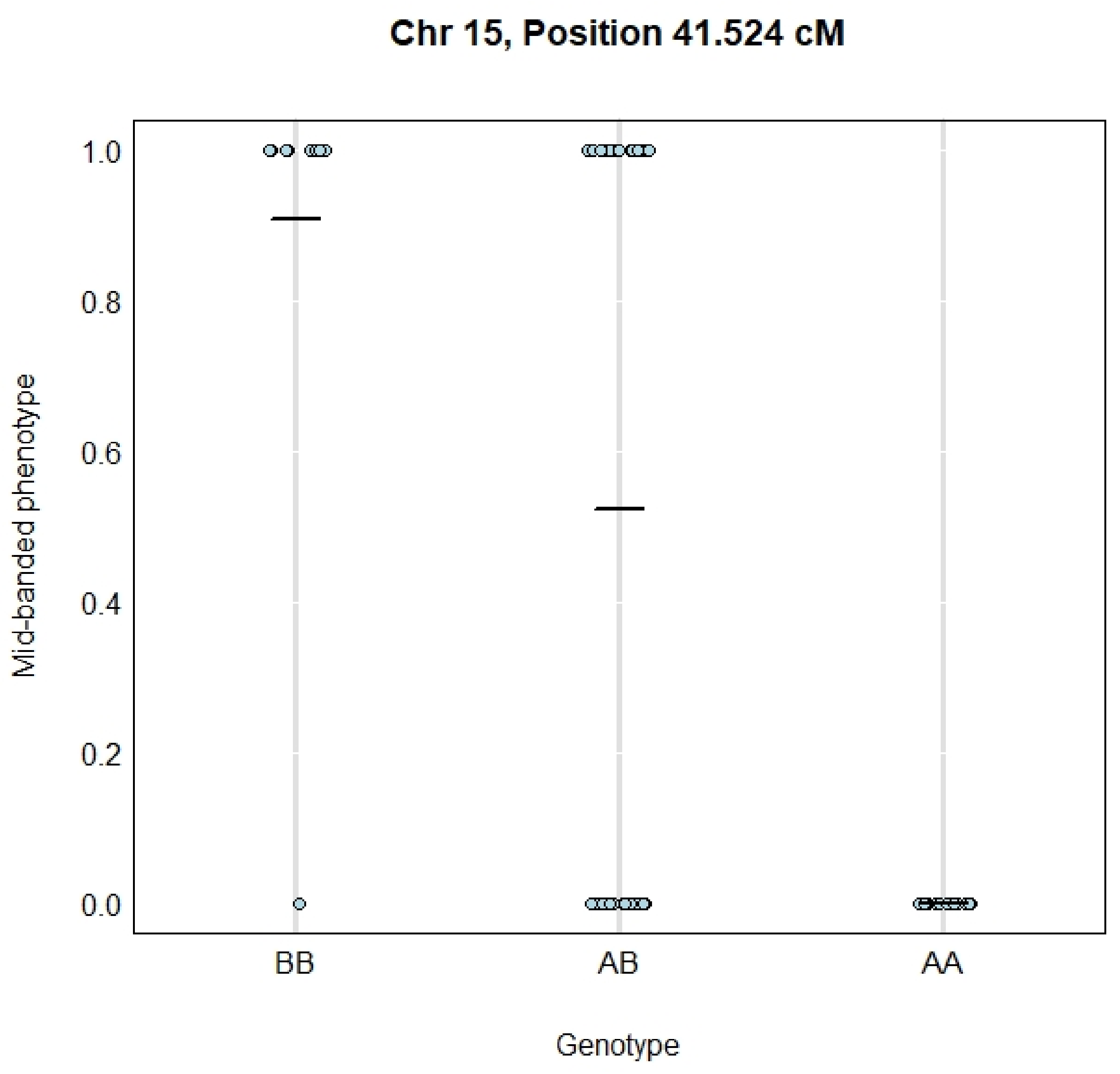

Chr 15, Position 41.524 cM
Mid-banded phenotype
1.0
0.8
0.6
0.4
0.2
0.0
BB
AB
AA
Genotype

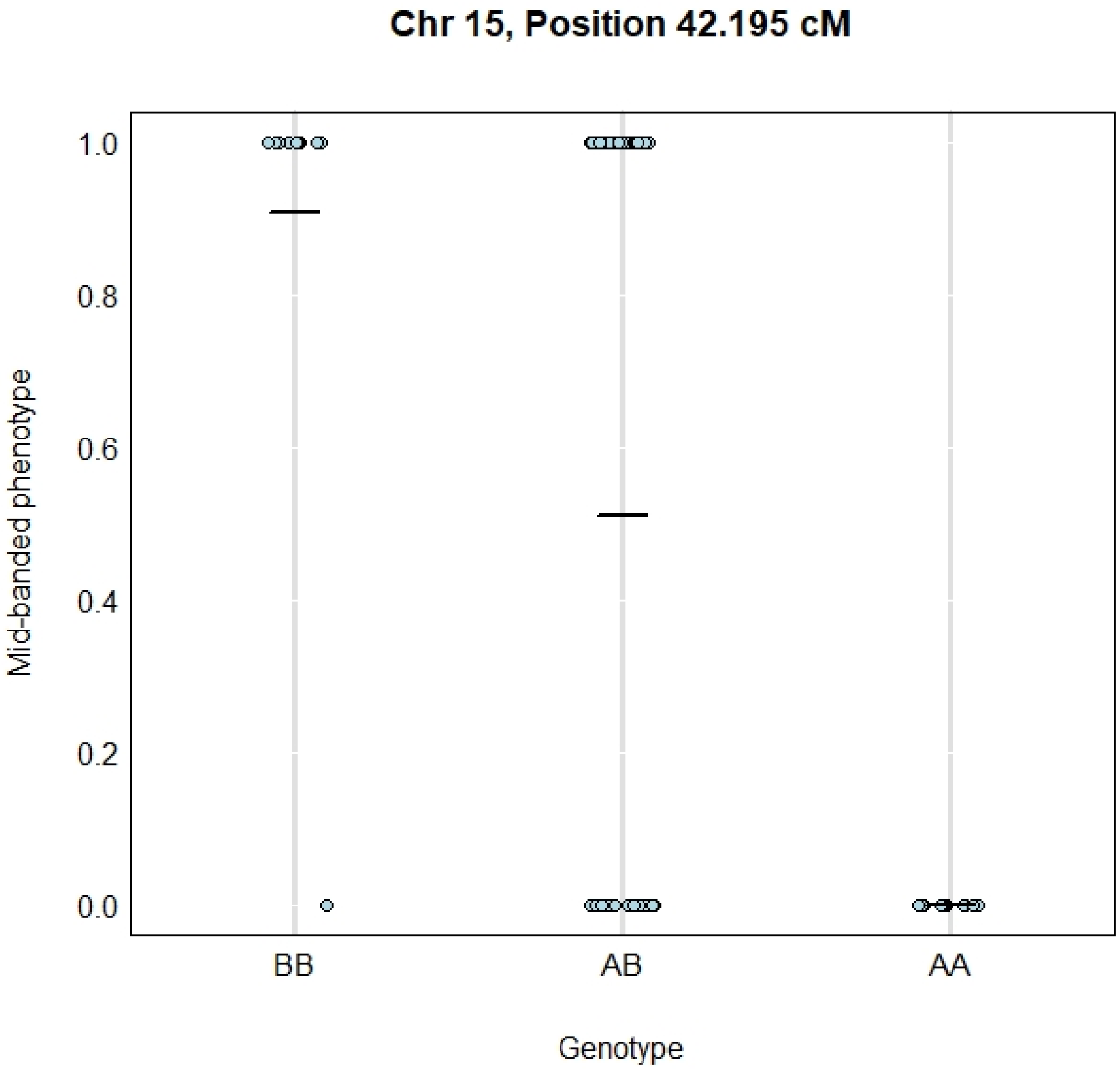

Chr 15, Position 42.195 cM
1.0
0.8
0.6
0.4
0.2
0.0
Mid-banded phenotype
BB
AB
AA
Genotype

Chr 15, Position 42.867 cM

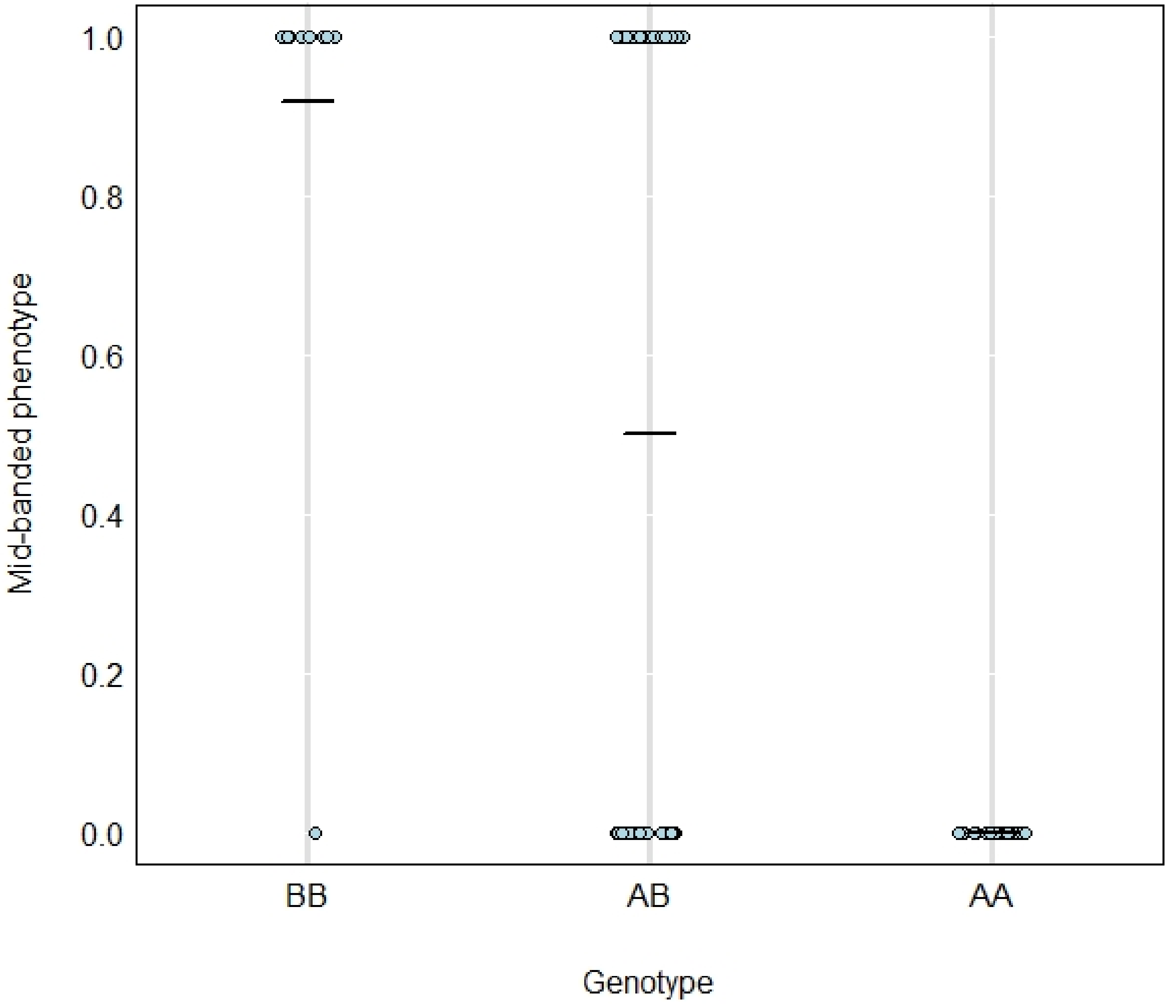

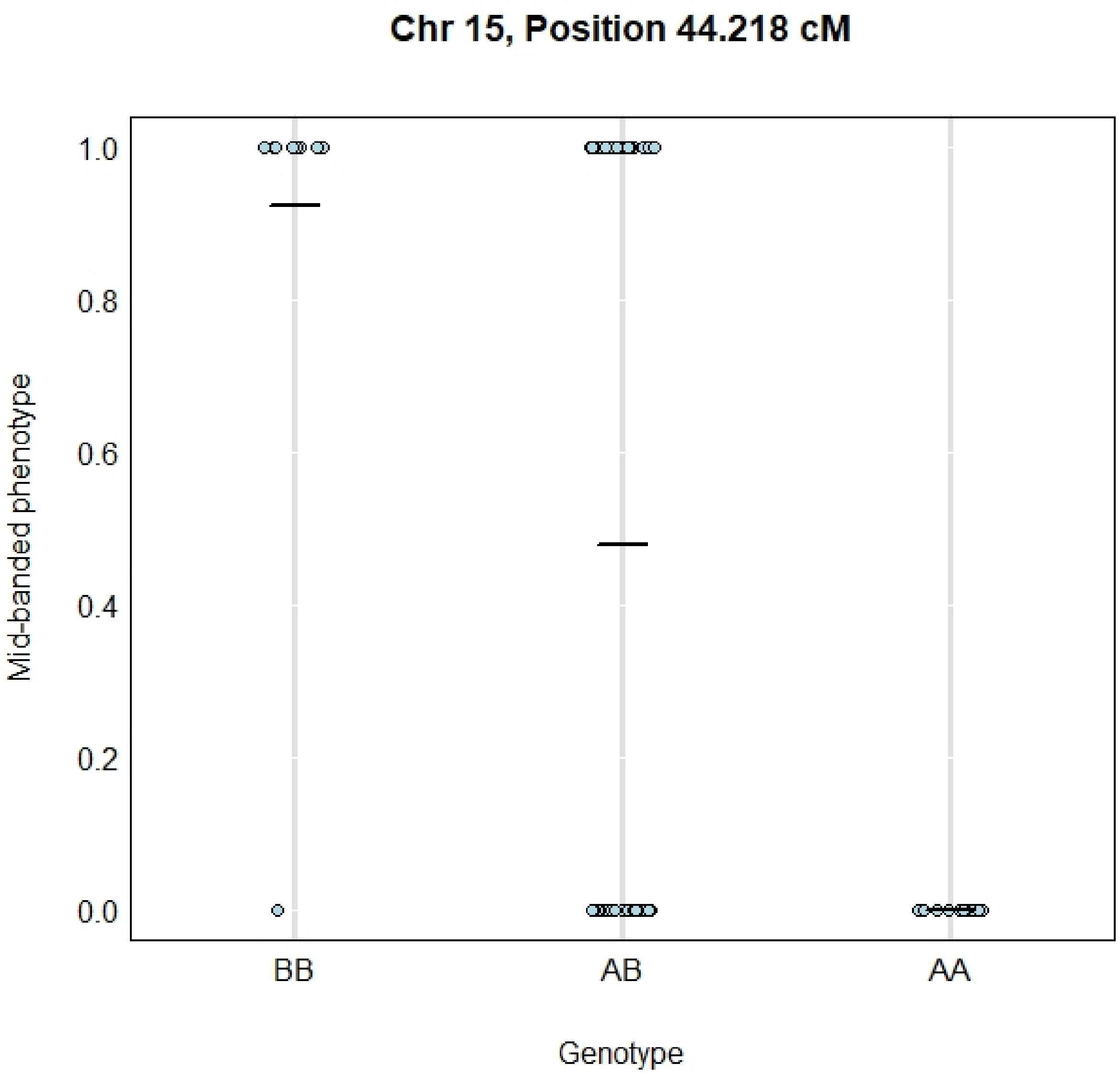
Chr 15, Position 44.218 cM
1.0
0.8
0.6
0.4
0.2
0.0
Mid-banded phenotype
BB
AB
AA
Genotype
