# Supplementary figures and images for: Fine mapping of the Cepaea nemoralis shell colour and mid-banded loci using a high-density linkage map
Source: Heredity (Edinb). 2023 Sep 27;131(5-6):327–37. doi: 10.1038/s41437-023-00648-z (PMC10673960; doi:10.1038/s41437-023-00648-z)

LG 1

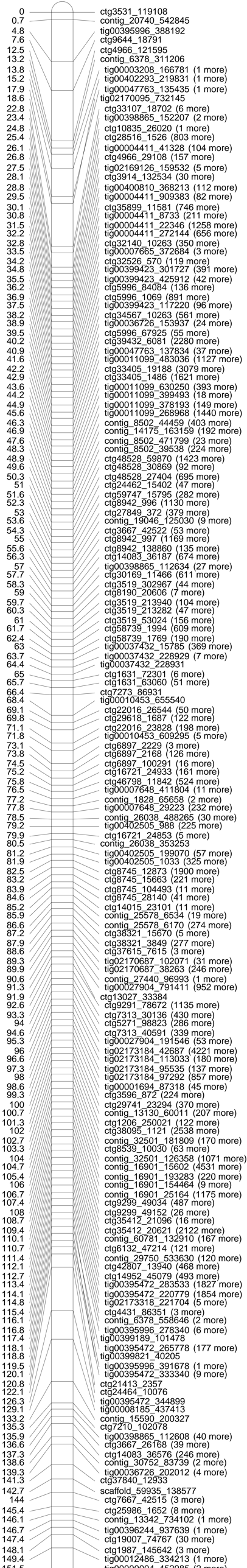

LG 2

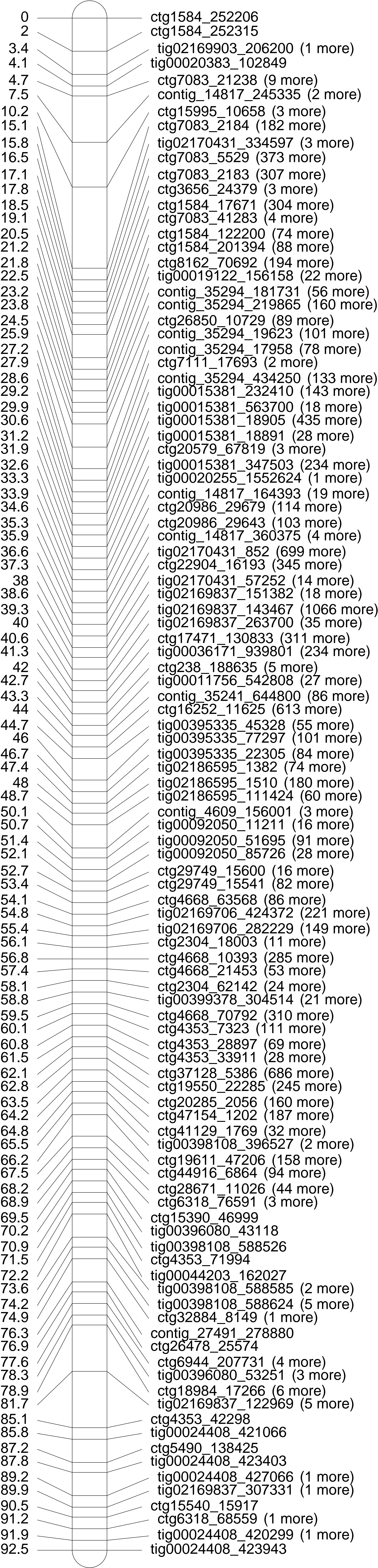

LG 3

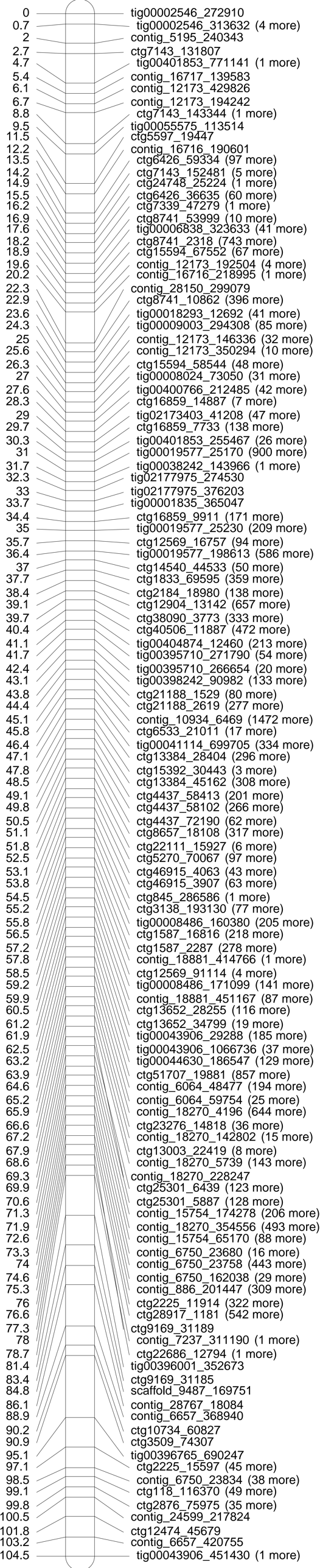

LG 4

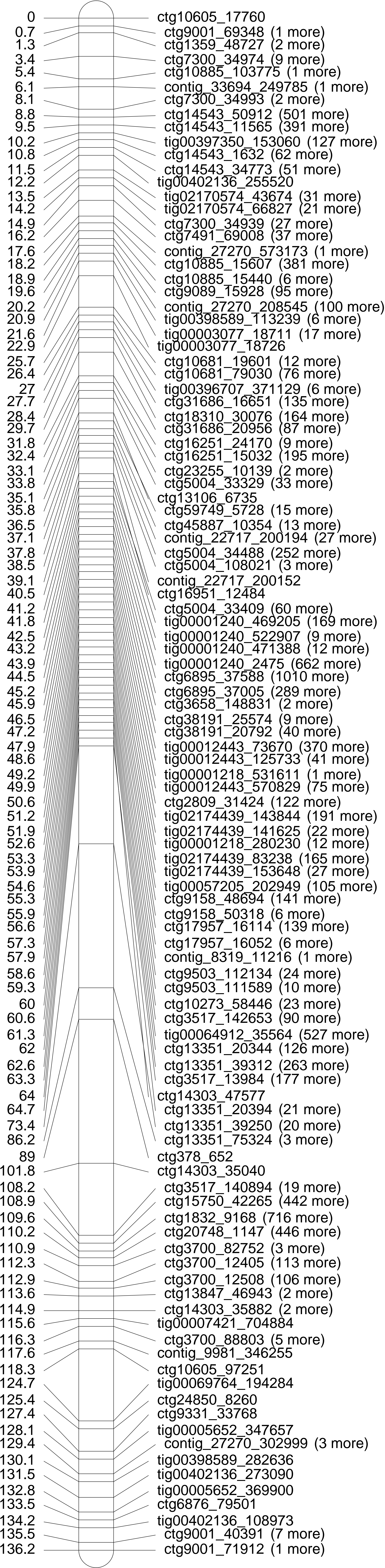

LG 5

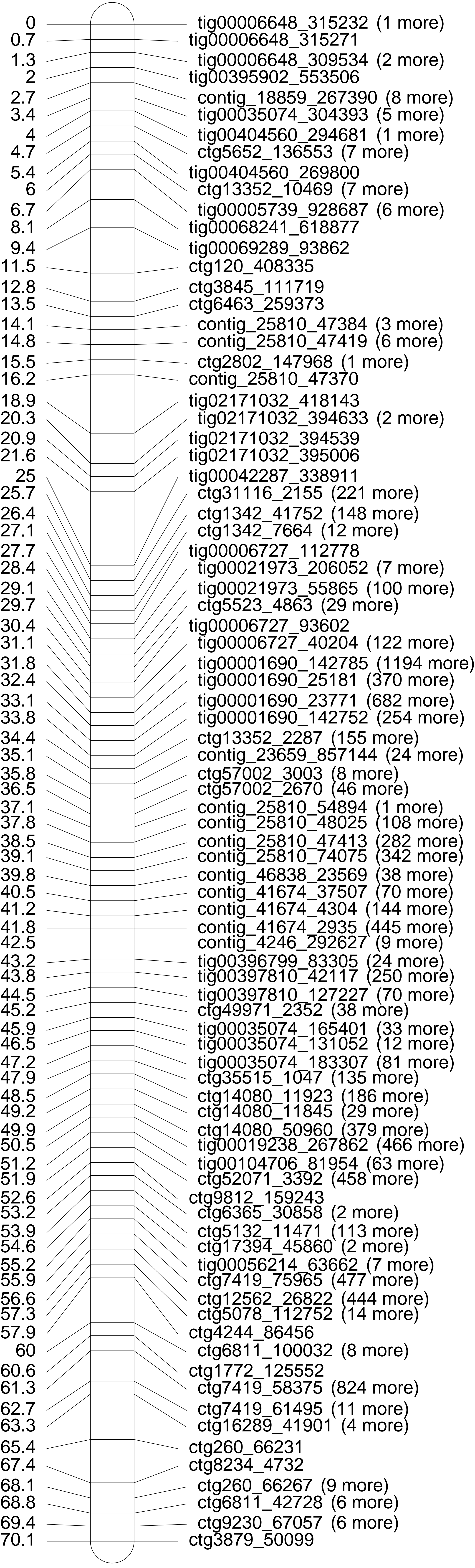

LG 6

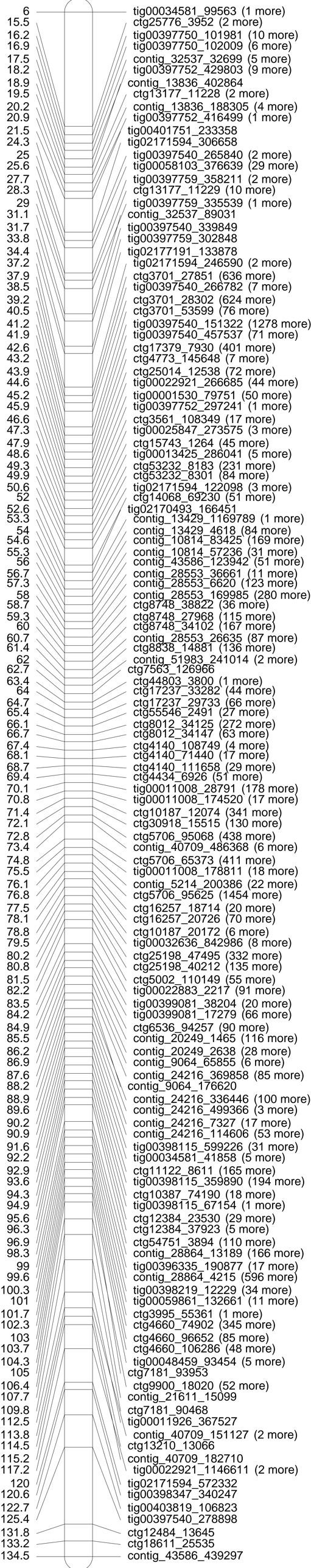

LG 7

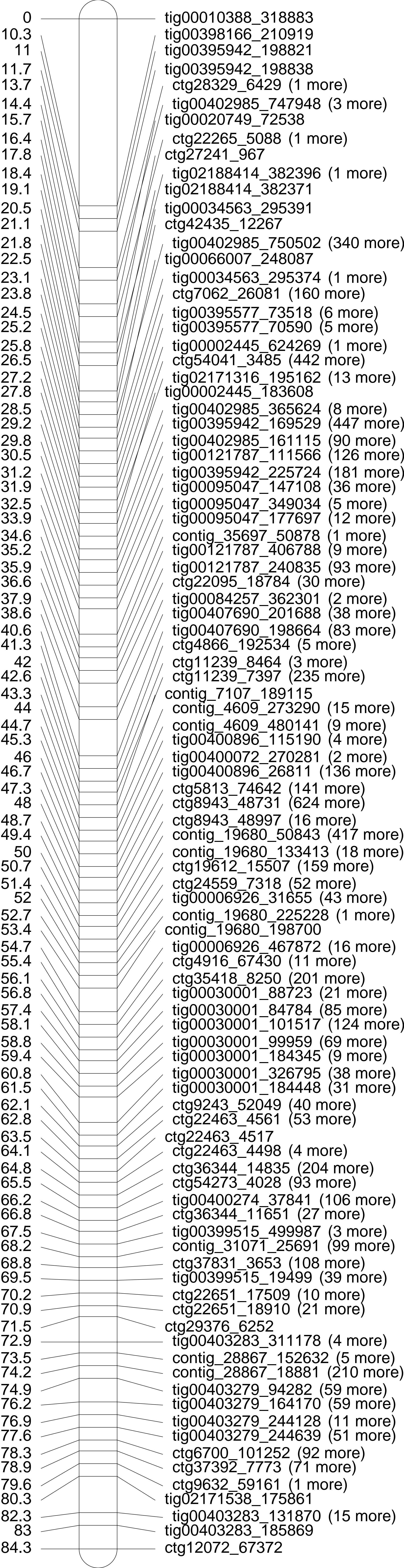

LG 8

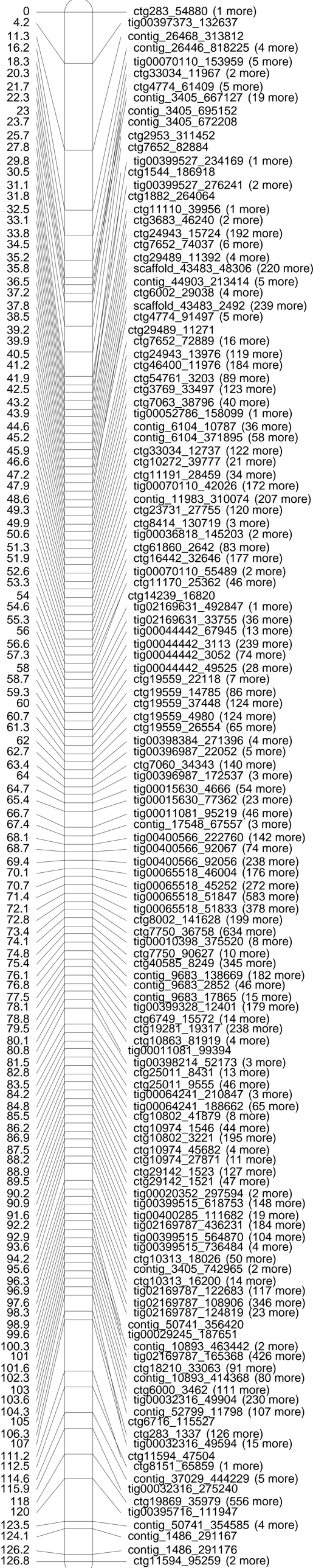

LG 9

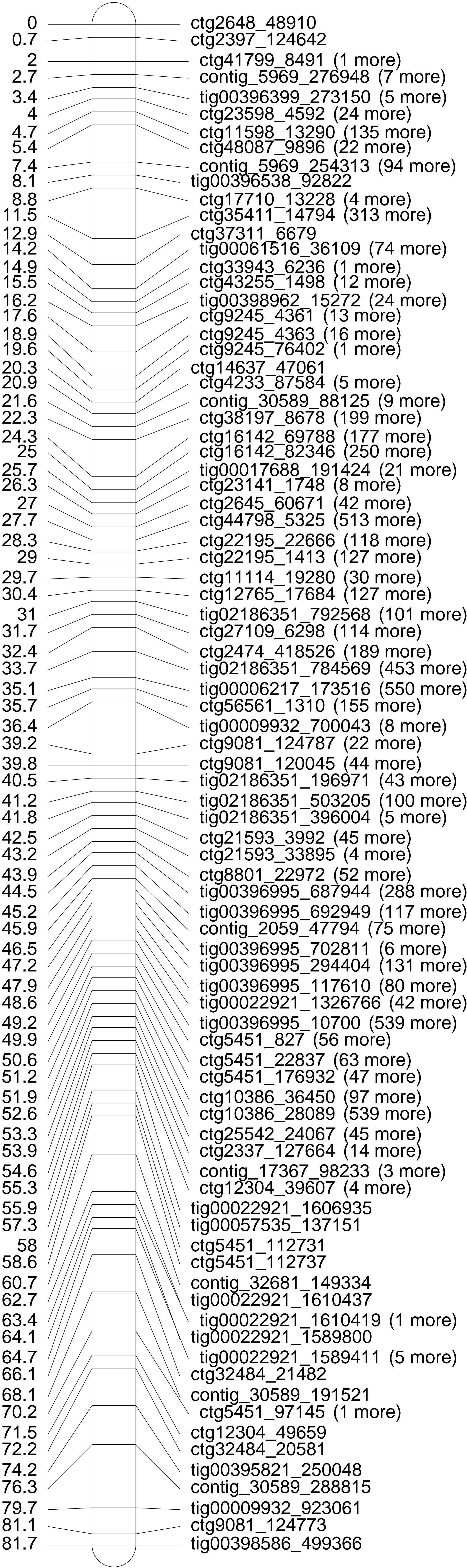

LG 10

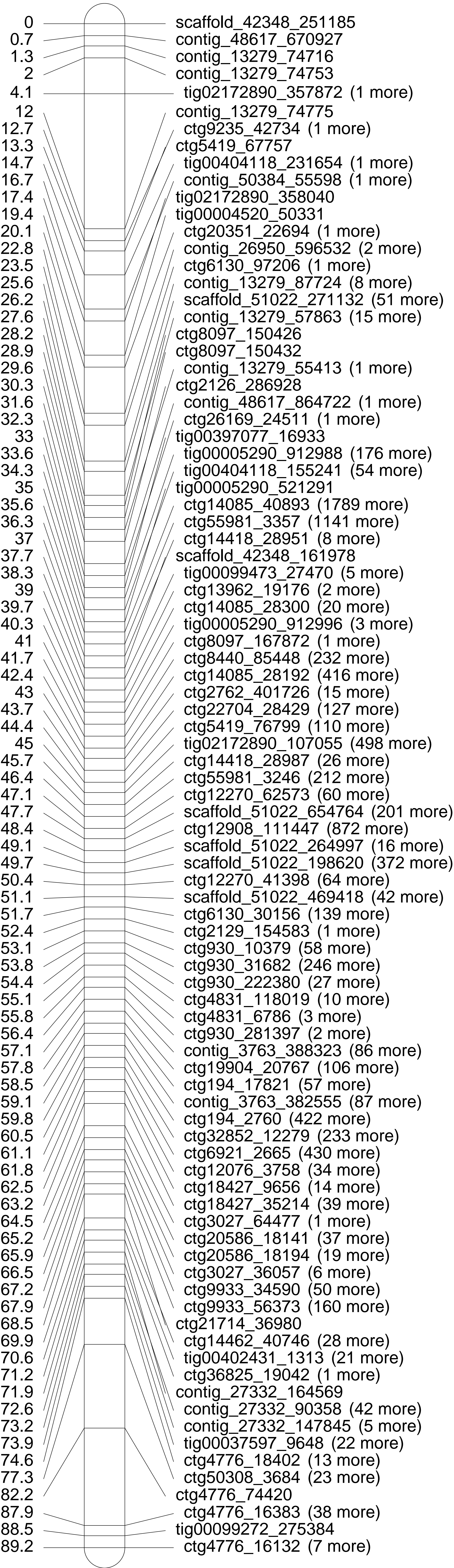

LG 11

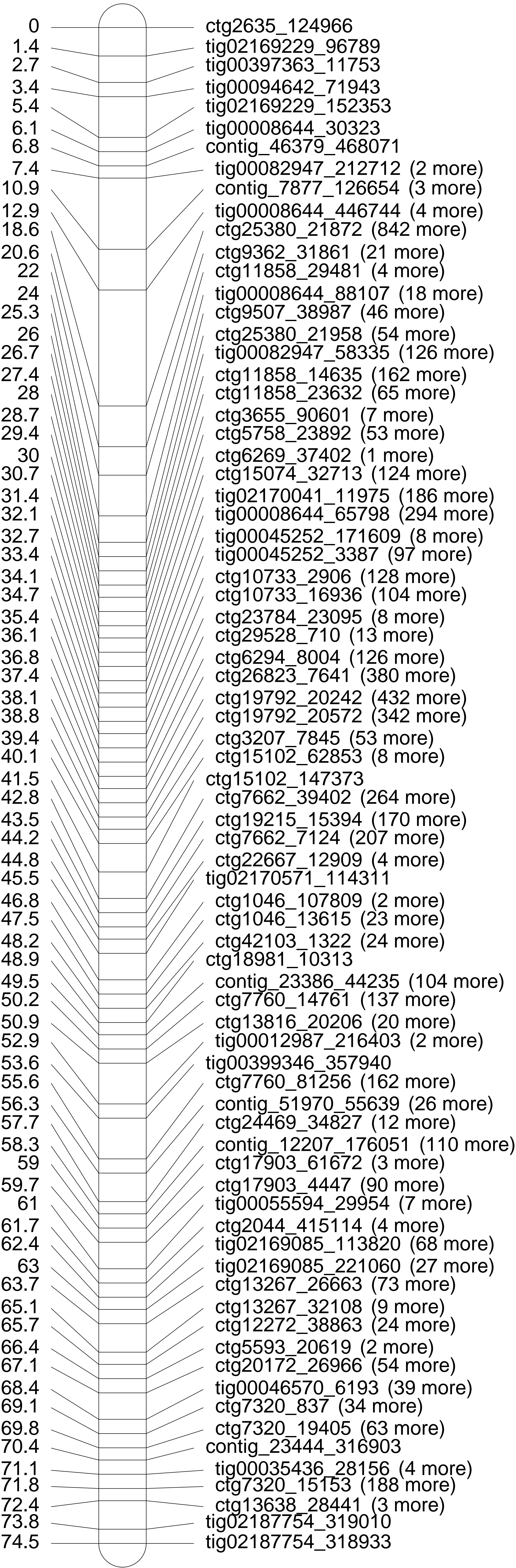

LG 12

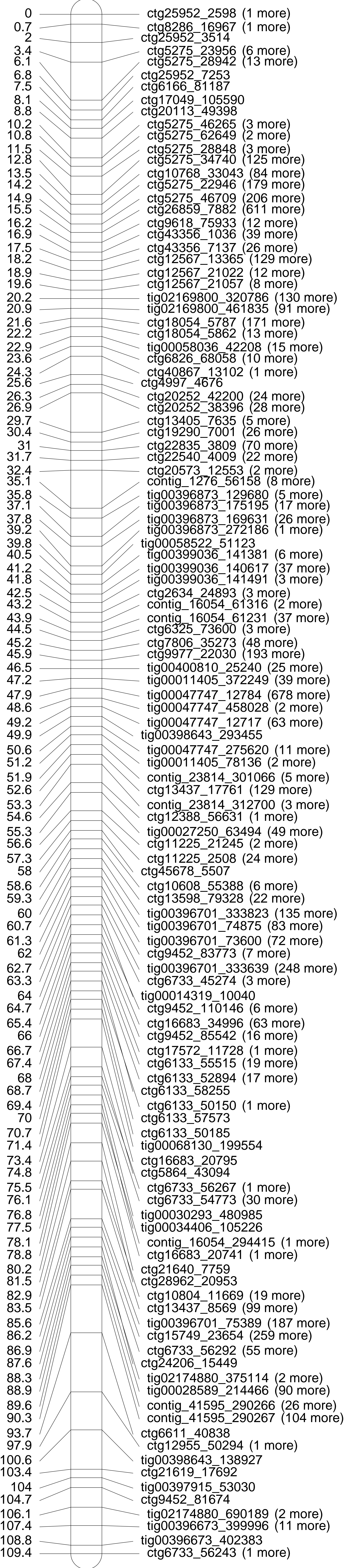

LG 13

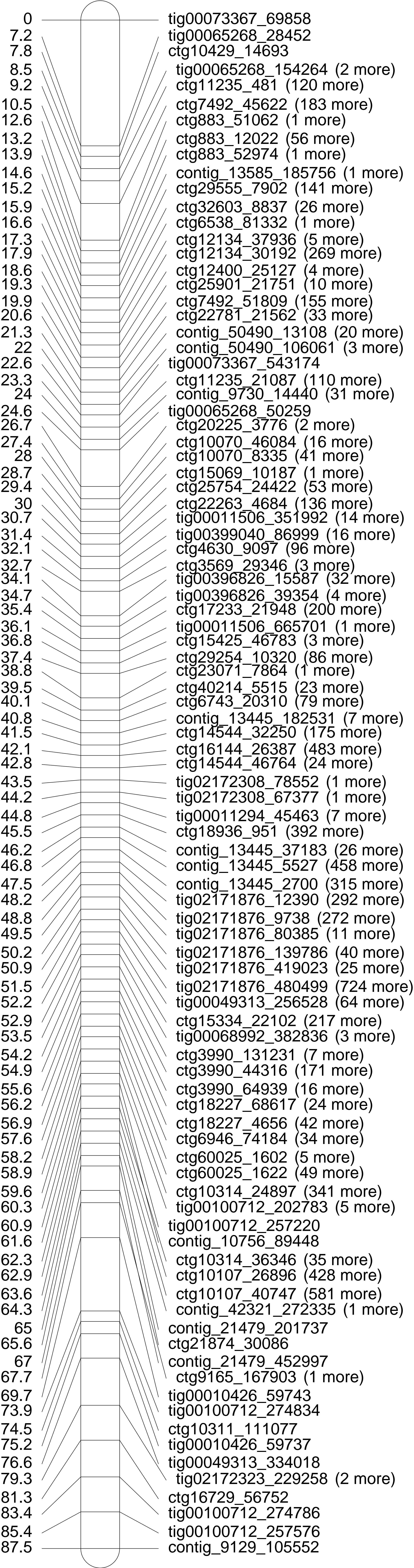

LG 14

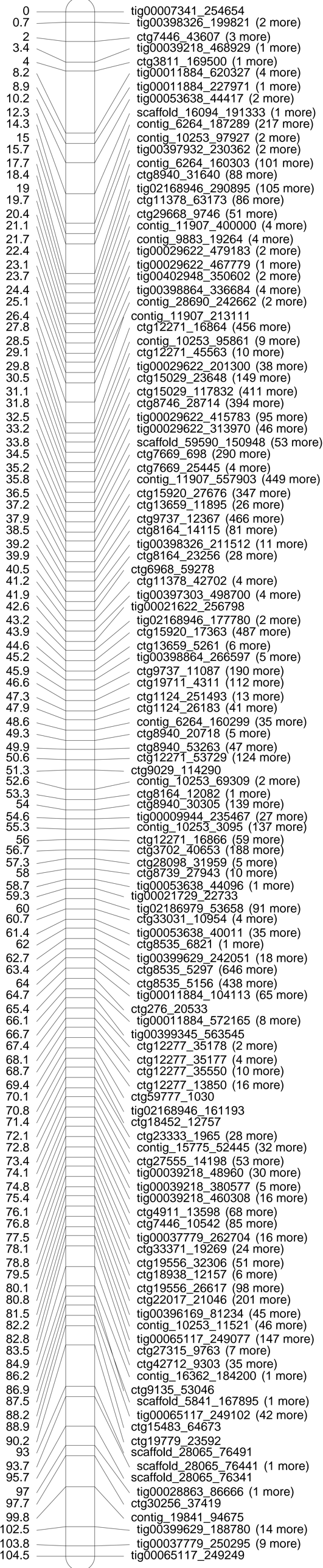

LG 15

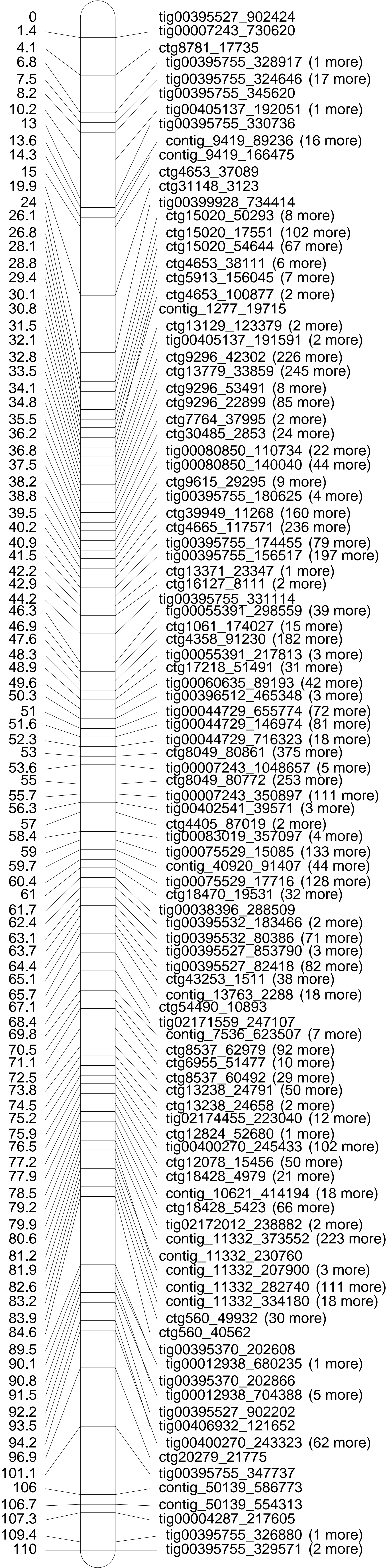

LG 16

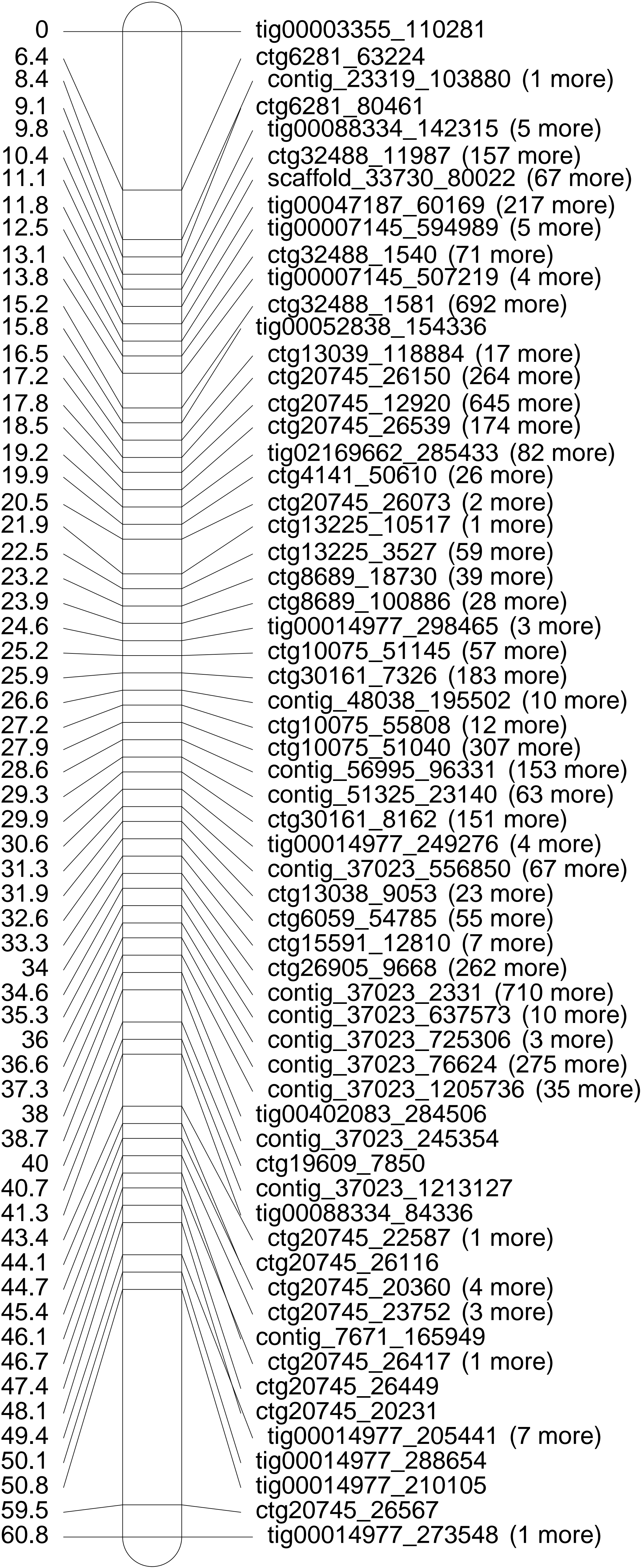

LG 17

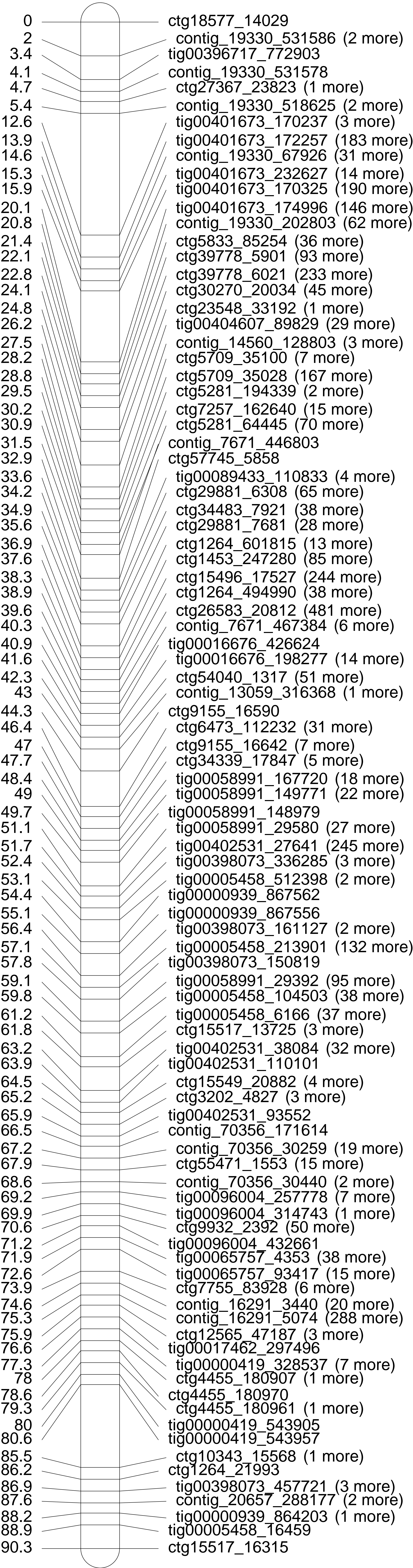

LG 18

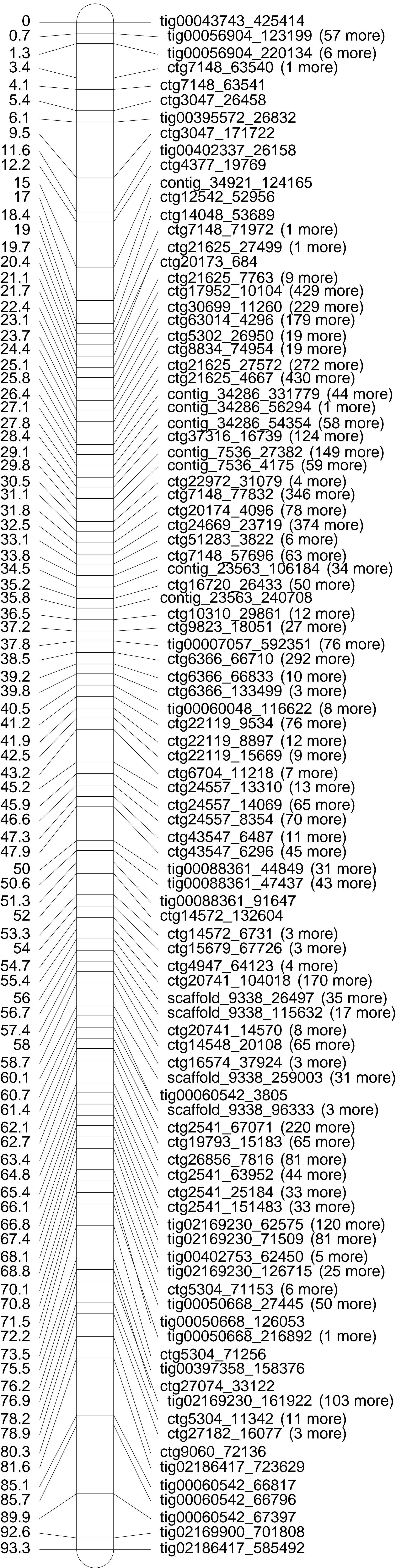

LG 19

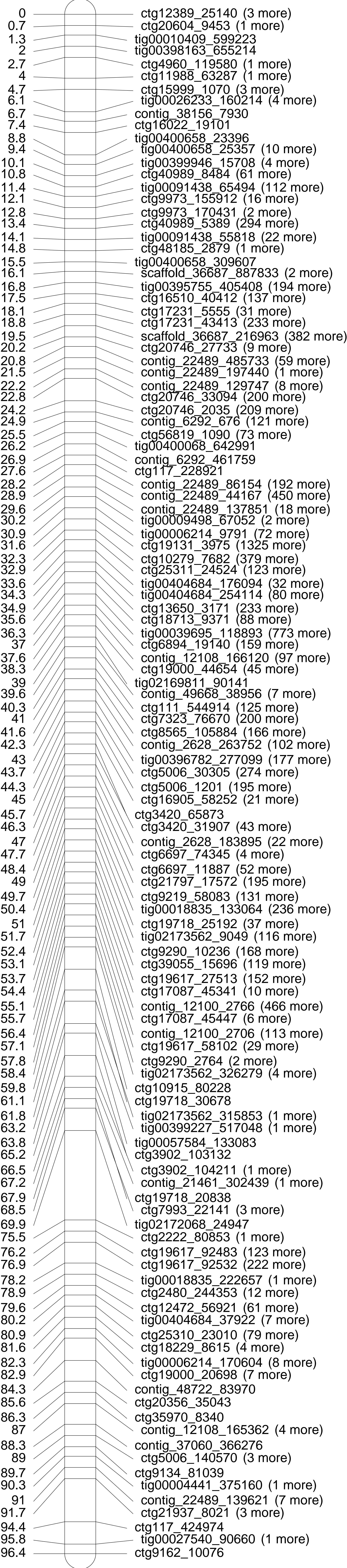

LG 20

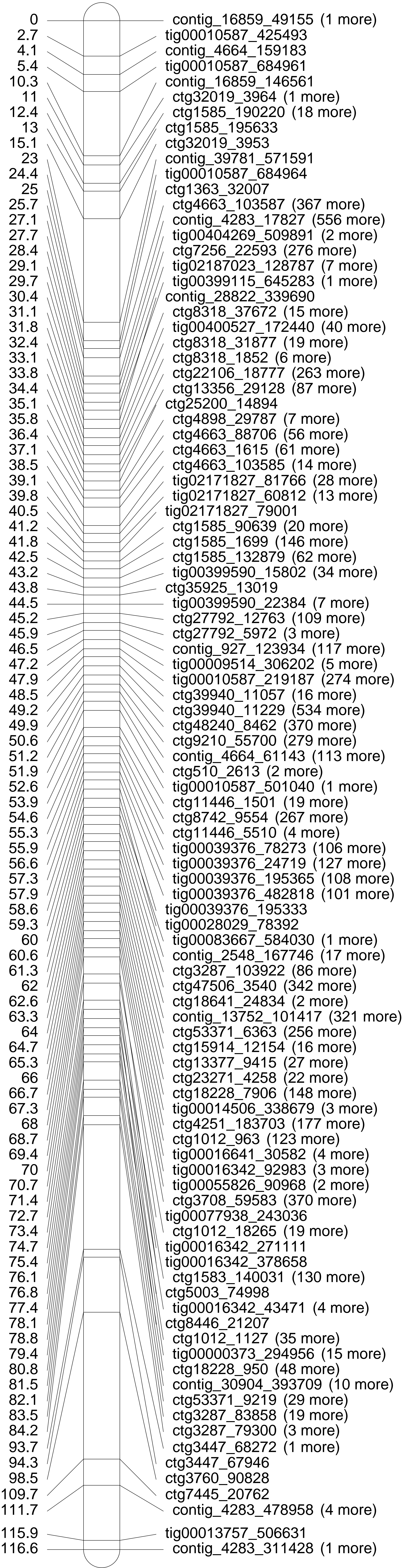

LG 21

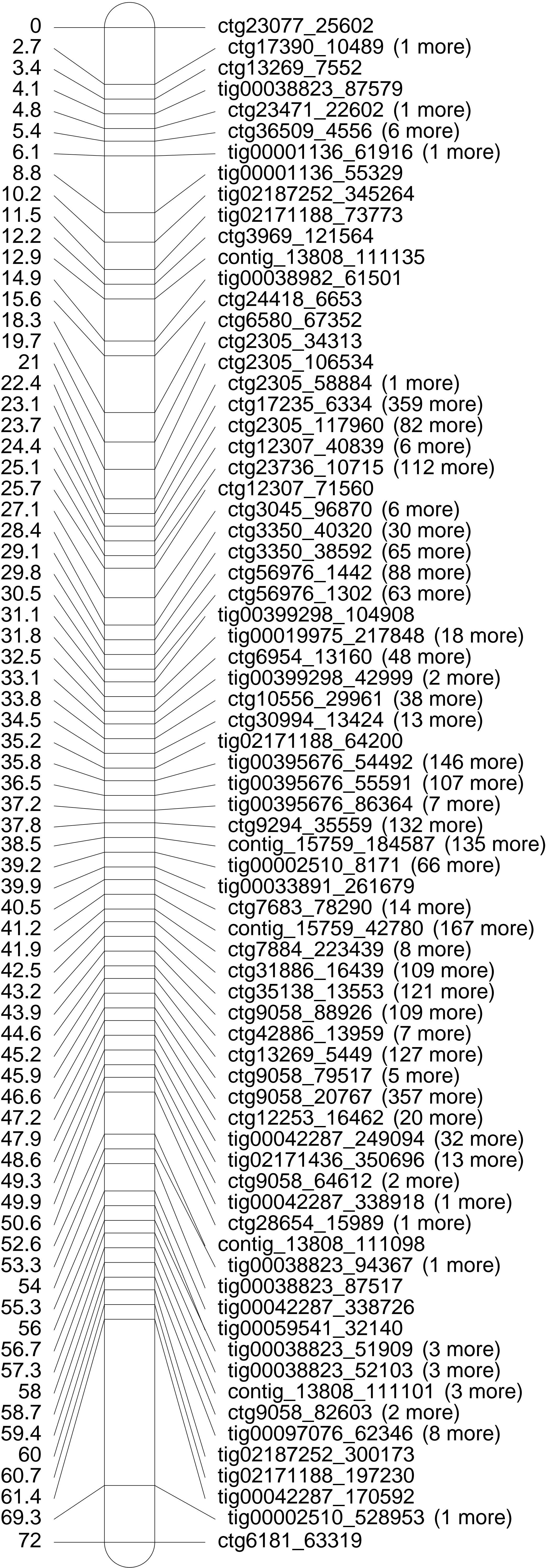

LG 22

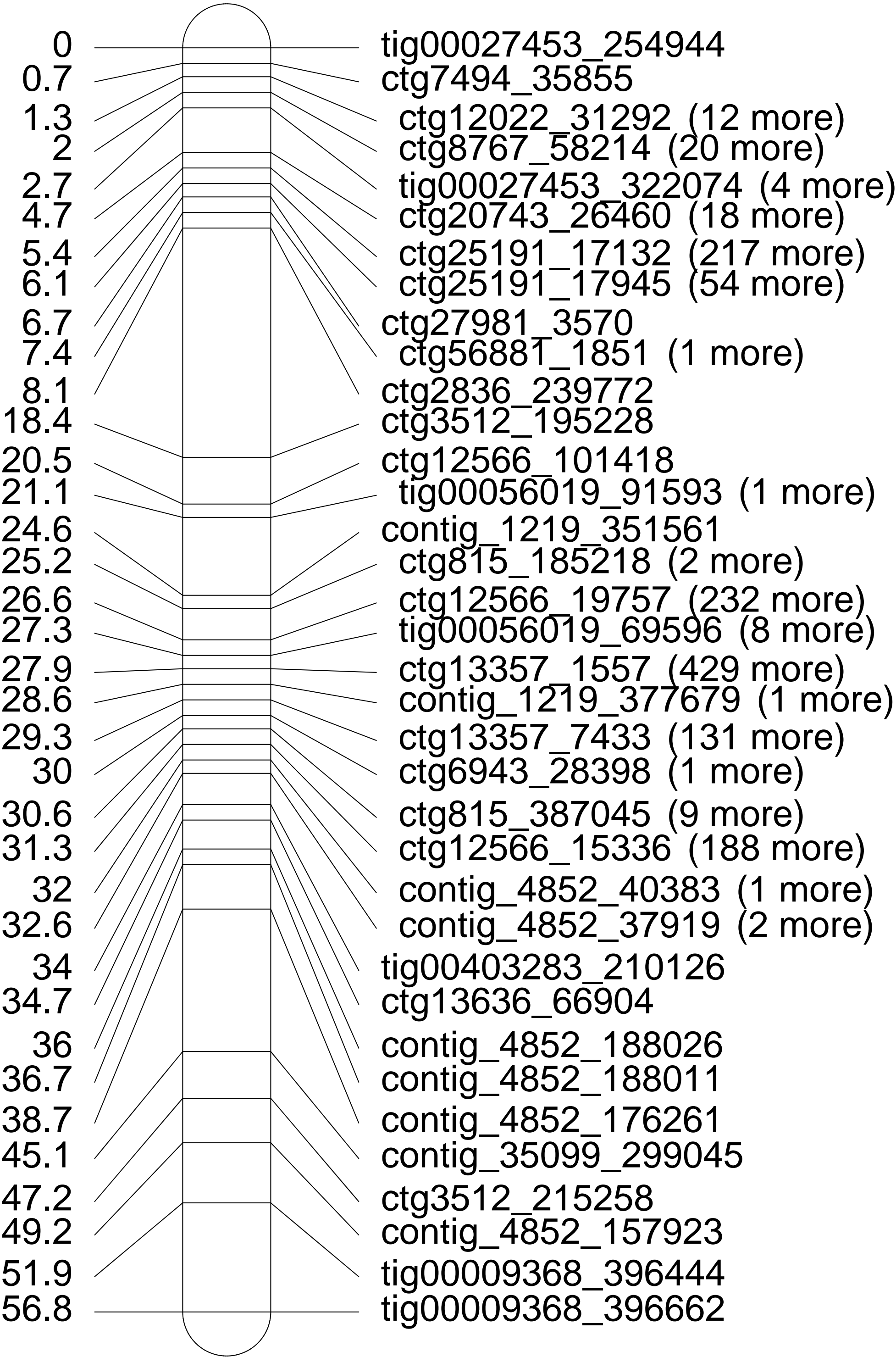

Supplement: Supplementary file 2 — Figure S1 [file 41437_2023_648_MOESM2_ESM.pdf]
